# Substitution of Met-38 to Ile in γ-synuclein found in two patients with amyotrophic lateral sclerosis induces aggregation into amyloid

**DOI:** 10.1073/pnas.2309700120

**Published:** 2024-01-03

**Authors:** Liam D. Aubrey, Natalia Ninkina, Sabine M. Ulamec, Natalia Y. Abramycheva, Eftychia Vasili, Oliver M. Devine, Martin Wilkinson, Eilish Mackinnon, Galina Limorenko, Martin Walko, Sarah Muwanga, Leonardo Amadio, Owen M. Peters, Sergey N. Illarioshkin, Tiago F. Outeiro, Neil A. Ranson, David J. Brockwell, Vladimir L. Buchman, Sheena E. Radford

**Affiliations:** ^a^Astbury Centre for Structural Molecular Biology, School of Molecular and Cellular Biology, Faculty of Biological Science, University of Leeds, Leeds LS2 9JT, United Kingdom; ^b^School of Biosciences, Cardiff University, Cardiff CF10 3AX, United Kingdom; ^c^Department of Pharmacology and Clinical Pharmacology, Belgorod State National Research University, Belgorod 308015, Russian Federation; ^d^Laboratory of Neurobiology and Tissue Engineering, Brain Science Institute, Research Center of Neurology, Moscow 125367, Russia; ^e^Laboratory of Molecular and Chemical Biology of Neurodegeneration, Institute of Bioengineering, School of Life Sciences, Ecole Polytechnique Fédérale de Lausanne, Lausanne CH-1015, Switzerland; ^f^Astbury Centre for Structural Molecular Biology, School of Chemistry, University of Leeds, Leeds LS2 9JT, United Kingdom; ^g^Department of Experimental Neurodegeneration, Center for Biostructural Imaging of Neurodegeneration, University Medical Center Göttingen, Göttingen 37075, Germany; ^h^Max Planck Institute for Multidisciplinary Sciences, Goettingen 37075, Germany; ^i^Translational and Clinical Research Institute, Faculty of Medical Sciences, Newcastle University, Newcastle Upon Tyne NE2 4HH, United Kingdom; ^j^Scientific employee with a honorary contract at Deutsches Zentrum für Neurodegenerative Erkrankungen, Göttingen 37075, Germany

**Keywords:** γ-synuclein, ALS, aggregation, amyloid, oligomers

## Abstract

α-, β-, and γ-Synuclein are intrinsically disordered proteins implicated in physiological processes in the nervous system of vertebrates. α-synuclein (αSyn) is the amyloidogenic protein associated with Parkinson’s disease and certain other neurodegenerative disorders. Intensive research has focused on the mechanisms that cause αSyn to form amyloid structures, identifying its NAC region as being necessary and sufficient for amyloid assembly. Recent work has shown that a 7-residue sequence (P1) is necessary for αSyn amyloid formation. Although γ-synuclein (γSyn) is 55% identical in sequence to αSyn and its pathological deposits are also observed in association with neurodegenerative conditions, γSyn is resilient to amyloid formation in vitro. Here, we report a rare single nucleotide polymorphism (SNP) in the *SNCG* gene encoding γSyn, found in two patients with amyotrophic lateral sclerosis (ALS). The SNP results in the substitution of Met38 with Ile in the P1 region of the protein. These individuals also had a second, common and nonpathological, SNP in *SNCG* resulting in the substitution of Glu110 with Val. In vitro studies demonstrate that the Ile38 variant accelerates amyloid fibril assembly. Contrastingly, Val110 retards fibril assembly and mitigates the effect of Ile38. Substitution of residue 38 with Leu had little effect, while Val retards, and Ala increases the rate of amyloid formation. Ile38 γSyn also results in the formation of γSyn-containing inclusions in cells. The results show how a single point substitution can enhance amyloid formation of γSyn and highlight the P1 region in driving amyloid formation in another synuclein family member.

## Significance

Understanding how synuclein proteins form amyloid in vitro and in cells is crucial to understand disease. Previous studies showed that the P1 region (residues 36–42) of α-synuclein controls amyloid formation. We here report a single nucleotide polymorphism in the P1 region of γ-synuclein (γSyn) (Met38 to Ile) found in two individuals with amyotrophic lateral sclerosis. Both individuals have a second polymorphism in the same gene (Glu110 to Val) that is commonly found in the general population. We show that Ile38-containing γSyn forms amyloid rapidly in vitro, while Met38 does not aggregate into amyloid and Val110 is protective, slowing aggregation. The results highlight the critical role of the P1 sequence in tipping the balance between a protein’s propensity for amyloid formation.

The synucleins (αSyn, βSyn, and γSyn) are a family of intrinsically disordered proteins (IDPs) with high expression levels in neuronal and certain non-neuronal tissues in all vertebrate species (1). αSyn is the most analyzed family member because of its association with synucleopathies such as Parkinson’s disease (PD), dementia with Lewy bodies (DLB), and multiple system atrophy (MSA). The self-assembly of αSyn into amyloid fibrils is known to be an important feature of these diseases, with the formation of oligomeric species (2, 3) and phase-separated states (4, 5) also contributing to fibril formation and proteotoxicity. Compared with αSyn, βSyn, and γSyn are relatively recalcitrant to amyloid fibril assembly. βSyn requires low pH, the addition of low concentrations of SDS, or the addition of metal ions (6–8), while γSyn requires extremely high protein concentrations or low pH for amyloid formation in vitro (9–11). Despite having a lower amyloid propensity than αSyn, pathological aggregates of γSyn have been detected in the nervous system of patients with several neurodegenerative diseases (12–16). The aggregation of γSyn has been suggested to contribute to the development of motor neuron pathology in amyotrophic lateral sclerosis (ALS), as aggregated γSyn was found within the descending axons of the corticospinal tract of patients (17) and overexpression of γSyn in neurons of transgenic mice causes middle-age-onset progressive motor neuron pathology that recapitulates many key characteristics of ALS (18, 19).

**Fig. 1.**
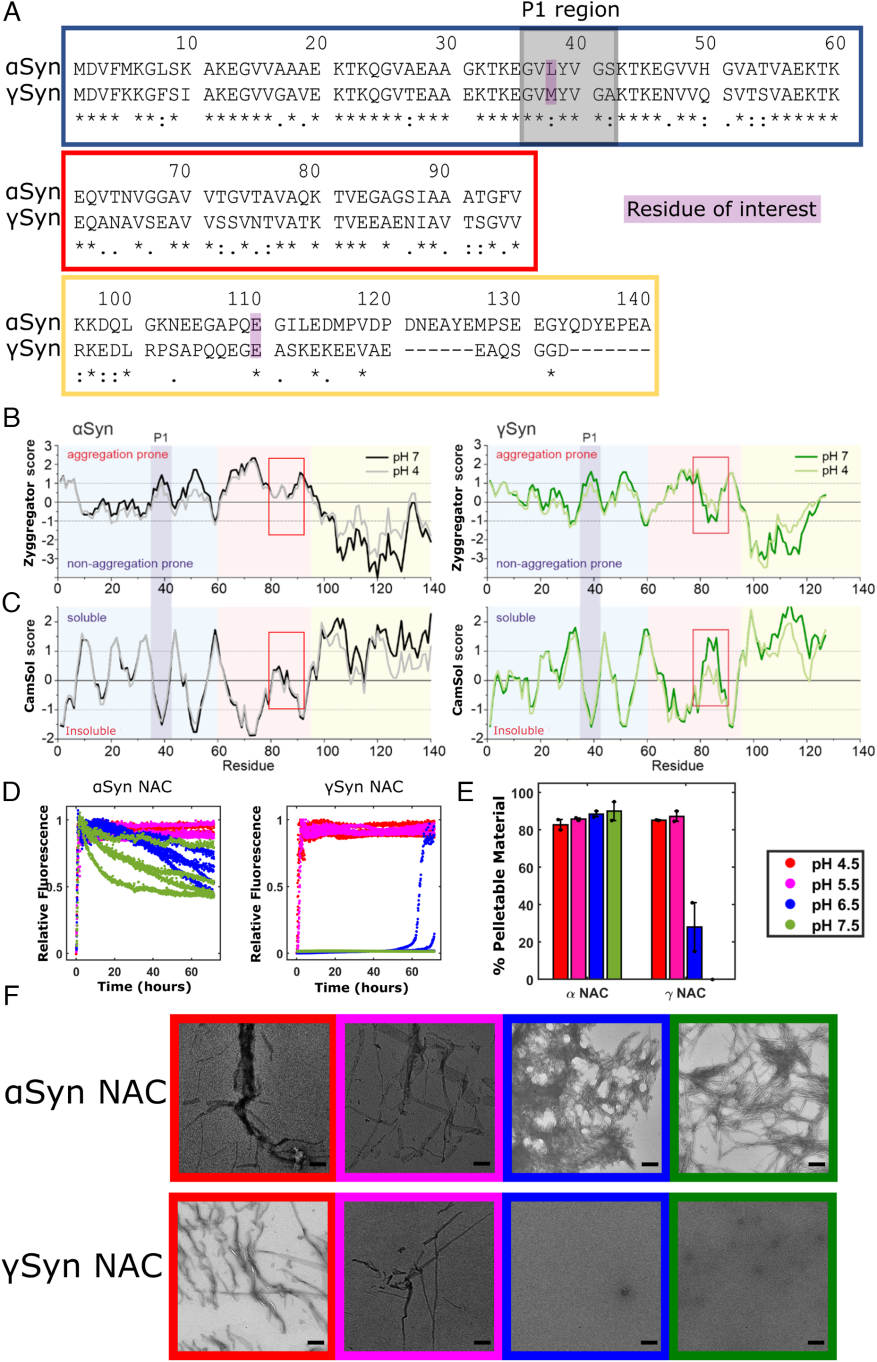
The γSyn NAC peptide does not form amyloid at neutral pH. (*A*) Sequence alignment [UniProt align tool (27)] of human αSyn and γSyn, highlighting the differences between the two paralogues. An “*” (asterisk) indicates identical residues at that position, “:” (colon) indicates conservation between groups of strongly similar properties—scoring > 0.5 in the Gonnet PAM 250 matrix (28), “.” (period) indicates conservation between groups of weakly similar properties—scoring ≤ 0.5 in the Gonnet PAM 250 matrix, and "-" (hyphen) represents a missing residue. Boxes show the N-terminal (blue), NAC (red), and the C-terminal regions (yellow). Residues of interest for this study (38 and 110) are highlighted in purple, and the P1 region is highlighted in gray. (*B*) Predicted aggregation propensity (Zyggregator score) (29) of αSyn (*Left*) and γSyn (*Right*) at pH 4.0 [gray (αSyn), light green (γSyn)] and pH 7.0 [black (αSyn), dark green (γSyn)]. Aggregation-prone regions have a positive Zyggregator score, whereas aggregation-resilient regions show negative values. N-terminal, NAC, C-terminal, and P1 regions are colored according to (*A*). (*C*) Predicted solubility using CamSol (30) of αSyn (*Left*) and γSyn (*Right*) at pH 4.0 and pH 7.0, colored as in (*B*). Soluble regions have a positive CamSol score, whereas insoluble regions show negative values. The red box highlights the different solubility and aggregation propensity of the C-terminal segment (residues 80–95) of αSyn NAC and γSyn NAC. (*D*) Amyloid assembly kinetics (ThT fluorescence) of 80 µM NAC peptide from αSyn (*Left*) and γSyn (*Right*) at pH 4.5 (red), pH 5.5 (pink), pH 6.5 (blue), or pH 7.5 (green). Note that no fibrils (zero ThT fluorescence) result for γSyn-NAC at pH 7.5. Reactions were performed at 37 °C with shaking at 600 rpm in the presence of a Teflon bead. (*E*) Differential pelleting assay demonstrating the relative amount of each peptide that was found in the pellet after centrifugation at 100,000*g* for 30 min. (*F*) Negative stain EM images taken at the end point of the assay, with the box outline color indicating the pH. (Scale bar, 200 nm.)

The difference in amyloidogenicity of α- and γSyn has been rationalized, at least in part, by changes in the central non amyloid β-component (NAC) region of their sequences (Fig. 1*A*). In αSyn, NAC (residues 61–95) is predicted to be highly insoluble and aggregation prone (20) and is required for fibril assembly in vitro and in cells (21–23). NAC forms part of the highly ordered core of all αSyn fibril structures solved to near-atomic resolution to date (24, 25) [with the exception of fibrils formed in the presence of Tau monomers in which residues 37–79 are found in the fibril core (26)]. Supporting the importance of NAC in amyloid formation, the absence of 11 residues in the NAC region of βSyn (relative to αSyn) ablates its ability to form amyloid under most conditions (6–8). Interestingly, while γSyn retains a NAC region, containing a 15-residue segment, known as the central NAC region (residues 65–79), which is predicted to have a high aggregation propensity, the C-terminal region of NAC (residues 80–95) is predicted to have low aggregation propensity and a higher predicted solubility than the equivalent region in αSyn (Fig. 1 *B* and *C*).

Despite NAC being both necessary and sufficient for αSyn fibril assembly, other regions of its sequence have been shown to alter the rate of fibril assembly in vitro and the propensity of the protein to form aggregates in vivo. For example, nine mutations associated with early-onset familial forms of PD [V15A (31), A30P/G (32, 33), E46K (34), H50Q (35), G51D (36), and A53T/E/V (37–39)] involve residues in the N-terminal region of αSyn. Extensive evidence has also shown that two regions in the N terminus, known as P1 (residues 36–42) and P2/pre-NAC (residues 47–57) (40, 41), play a crucial role in determining the ability of the protein to form amyloid. These regions also modulate αSyn-associated proteotoxicity in *Caenorhabditis elegans*, *Drosophila*, and primary cortical neurones (42–44). For example, single residue substitutions of residues Leu38 (with Met), or Tyr39 and Ser42 (with Ala) in αSyn, or deletion of the entire P1 region, can dramatically reduce the ability of the protein to form amyloid in vitro and reduce aggregation-associated proteotoxicity in *C. elegans* (41, 42). By contrast, deletions in the acidic C-terminal region enhance αSyn amyloid fibril assembly (45–47).

In contrast to αSyn, relatively little is known about the effects of amino acid substitutions on amyloid formation of γSyn and on disease etiology. The substitutions Met38Ala or Tyr39Ala in γSyn result in fewer SDS-resistant aggregates in the presence of dopamine (48). However, the effect of these substitutions on the rate of amyloid formation has not been investigated. Here, we identified a rare *SNCG* variant that is present in two ALS patients but not in the control group from the same population. This SNP results in the substitution of Met38 with Ile in the encoded protein. An unusually early onset of the disease in both patients suggests that the substitution of M38 with Ile could contribute to faster development of severe neurodegenerative changes. To determine whether this substitution, which lies in the P1 region of γSyn, increases the aggregation potential of the protein, akin to the behavior observed for αSyn (41, 42), we studied amyloid formation of the Met38 and Ile38 γSyn variants in vitro, in cells, and in *Drosophila*. We show that the presence of Ile38 in γSyn results in a dramatic enhancement in fibril assembly in vitro while substitutions at this position with other aliphatic residues had different effects on amyloid formation. The results demonstrate that the sequence of P1 is crucial for aggregation of γSyn into amyloid, at least in vitro. Puncta formation in cells is enhanced by the M38I substitution, while proteotoxicity is not observed in the cells tested or in fly models. The results label the P1 region as an enticing target for the development of modulators of amyloid formation for disorders involving the aberrant self-assembly of both αSyn and γSyn proteins. Interestingly, a second polymorphism in *SNCG* was found in the individuals concerned, which results in substitution of Glu110 with Val and is commonly found in the general population. We found this second amino acid substitution to have a protective effect on amyloid formation in vitro, partially mitigating the effect of I38 and demonstrating that γSyn aggregation into amyloid is finely balanced by residues across its sequence.

## Results

### The NAC Region of γSyn Does Not Form Amyloid at Neutral pH.

αSyn and γSyn have 77, 51, and 13% sequence identity in their N-terminal, NAC, and C-terminal regions, respectively (Fig. 1*A*). Camsol [which predicts solubility (30)] and Zyggregator [which predicts amyloid propensity (29)] were used to explore how these sequence differences alter the predicted properties of the two proteins (Fig. 1 *B* and *C*). The N-terminal regions of αSyn and γSyn (residues 1–60) are predicted to have similar per-residue solubility and aggregation profiles, with the P1 (and P2) regions showing peaks in amyloid potential and low solubility scores for both proteins, consistent with their high sequence similarity (67 and 55%, respectively) in these regions. The major differences in sequence in the C-terminal regions of the two proteins result in little change in their predicted behaviors (both showing high solubility and low amyloid potential for this region). By contrast, large differences in predicted behavior are observed for residues 80–95 that form the C-terminal half of NAC. In αSyn, this region is predicted to be weakly soluble and weakly aggregation-prone, whereas these residues are predicted to be highly soluble and aggregation resilient in γSyn, especially at pH 7 as two glutamic acids (E84 and E86) are substituted into γSyn-NAC relative to αSyn-NAC [which has Gly at these positions (Fig. 1*A*)]. This could contribute to the observed differences in amyloid propensity of the two proteins (9–11), although differences in helical propensity of the full-length proteins have also been implicated (49).

To explore how changes in the sequence of NAC affect the ability of this region to form amyloid, peptides spanning NAC (residues 61–95) were synthesized (*Materials and Methods* and *SI Appendix*, Fig. S1), and amyloid fibril formation was monitored at different pH values using thioflavin T (ThT) fluorescence (Fig. 1*D*). Consistent with the predictions, the αSyn-NAC peptide rapidly assembled into amyloid fibrils at all pHs measured, with no clear lag time observed and half-times of 1 h or less at all pH values (*SI Appendix*, Table S1). Fibrils resulted almost as rapidly for γSyn-NAC at pH 4.5 and pH 5.5 (half-times of 0.7 h and 1.5 h, respectively, with some fibrils appearing at pH 6.5 (lag time > 60 h) and no fibrils at pH 7.5. Analysis of the aggregate yield by ultracentrifugation followed by HPLC of the soluble fraction and negative stain TEM of the whole sample confirmed the results observed by ThT (Fig. 1 *E* and *F*). Accordingly, little pelletable material (28%) was observed for γSyn-NAC at pH 6.5 with no fibril formation occurred for γSyn-NAC at pH 7.5, where the whole sample remained in the soluble fraction after ultracentrifugation. Such a marked difference in behavior of the NAC peptides could contribute to the corresponding differences in the amyloid potential of their parent proteins (9–11).

### Identification of a SNP Causing a Substitution in the P1 Region of γSyn.

Exon sequencing of the *SNCG* gene in cohorts of ALS patients and healthy controls from the Central European Russian population identified a single nucleotide G > A substitution in the first exon of the gene in two of 140 assessed ALS patients (MAF 0.00714). Both had young spinal onset (35 y for the male patient and 36 y for the female patient), no known family history of ALS, and no mutations in other genes associated with ALS etiology (SOD1, C9orf72, TDP43, FUS, and ANG). This genetic variant corresponding to the SNP rs148591902 has not been found in 265 healthy individuals from the same population and is rare in publicly available databases (e.g., MAF is 0.00024 for the European population and 0.00012 overall in gnomAD). This single nucleotide substitution leads to an amino acid substitution of Met38 for Ile located in the P1 region of γSyn. Both patients carrying this substitution also carried a previously described (50, 51) minor allele of the SNP rs9864 (MAF is 0.23-0.27) in all available databases for ALS patients and in the general population, that encodes valine (V) instead of glutamic acid (E) encoded by the major allele, at position 110 in the C-terminal region of γSyn. By using long-range PCR amplification followed by cloning of resulting fragments and sequencing of a number of individual clones, we showed that both patients carried one copy of *SNCG* encoding the common M38/E110 sequence (shown in Fig. 1*A*) and another copy encoding the rare I38/V110 variant of γSyn.

In the absence of the family history of the disease for both patients, we cannot annotate the M38I polymorphism as being directly causative or linked to the pathology of ALS. Nonetheless, the identification of a naturally occurring SNP in *SNCG* that results in a substitution in the P1 region of γSyn inspired us to investigate further its effect on amyloid assembly in vitro and in cells, especially given the precedence that amino acid changes in the same region of αSyn can have profound influence on amyloid formation of this closely related protein (41, 42). Hence, the following in vitro data were collected to assess the role of the P1 region in γSyn amyloid formation, rather than to attempt to directly relate the properties of the protein in vitro with ALS pathology.

### The Substitution M38I Enhances γSyn Amyloid Assembly.

**Fig. 2.**
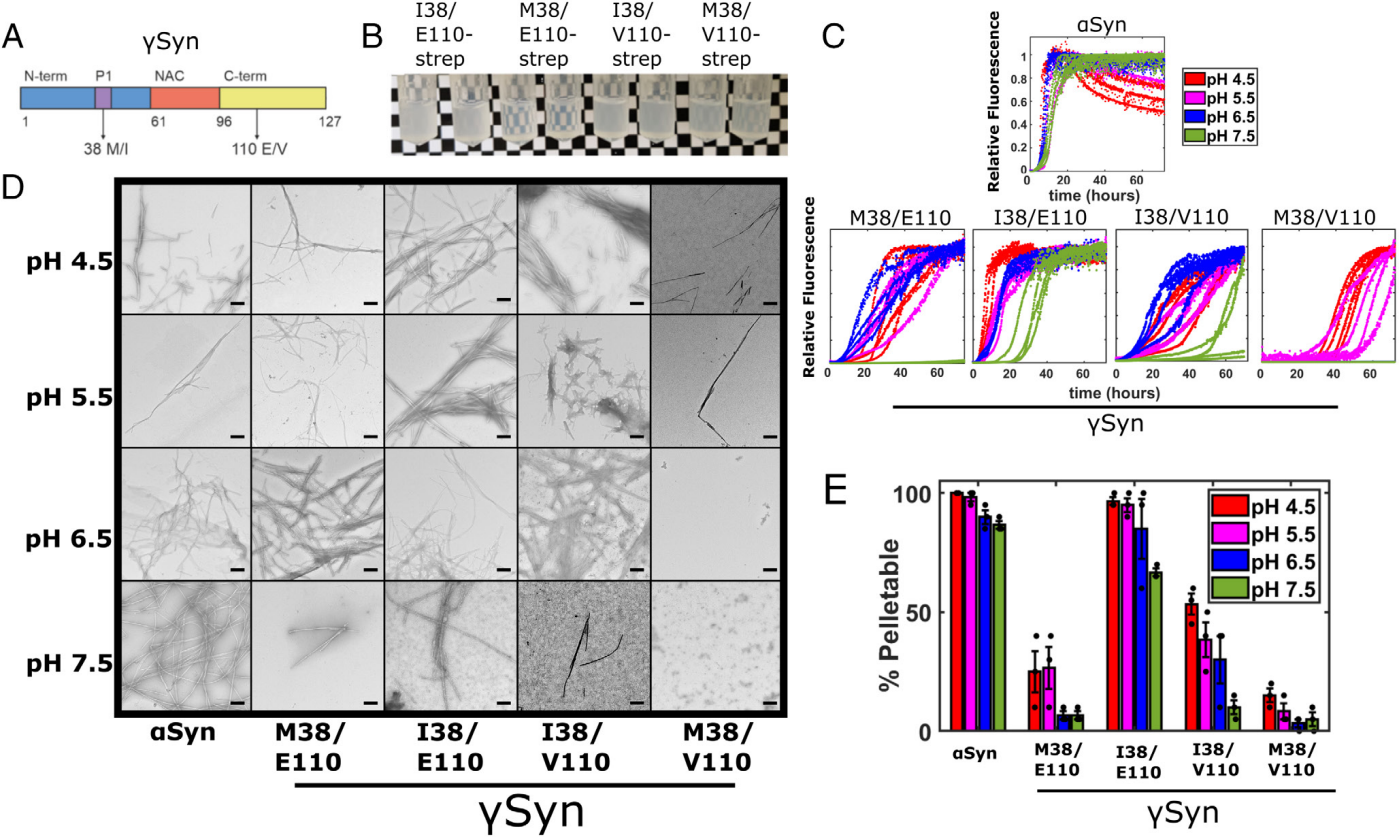
γSyn I38 variants result in more efficient amyloid fibril assembly. (*A*) Schematic showing the γSyn protein split into the N-terminal, NAC, and C-terminal regions highlighting the location of the two residues of interest. The previously discovered (50, 51) Glu/Val polymorphism at residue 110 in the C-terminal region (yellow) and the Met38Ile substitution in the P1 region (purple) within the N-terminal region (blue) are highlighted. Neither site is located within the NAC region (orange). (*B*) In-tube aggregation assay using 70 µM Strep-tagged γSyn variants. Images of tubes after 13 d of incubation at 37 °C in pH 8 buffer are shown. Variants containing the Ile38 are visibly more turbid, although some turbidity is observed for M38/V110. (C) ThT fluorescence of 80 μM αSyn and different γSyn variants monitored at 37 °C in 96-well plates. Assays were performed in a mixture of Tris and sodium acetate buffers at a total 20 mM ionic strength at pH 4.5 (red), 5.5 (pink), 6.5 (blue), or 7.5 (green). Four replicates of each are shown. Note that curves are normalized to a value of 1 if they reached a plateau, irrespective of the aggregate yield. Lag time and T_50_ values are shown in *SI Appendix*, Table S1. (*D*) Negative stain TEM images of samples at the end point of the ThT reactions. (Scale bar, 200 nm.) (*E*) Differential pelleting assay demonstrating the relative amount of protein that was found in the pellet after centrifugation at 100,000*g* for 30 min. The mean ± SD is shown (see also *SI Appendix*, Table S1).

Given that the P1 region (including residue 38) is known to control αSyn fibril assembly (41, 42), we hypothesized that the substitution of Met38 with Ile could enhance amyloid formation of γSyn [αSyn has Leu at residue 38 (Fig. 1*A*)]. To test this hypothesis, four variants were expressed recombinantly in *Escherichia coli*, purified, and analyzed to deconvolute the effects of the amino acid substitutions found at positions 38 (Met/Ile) and 110 (Glu/Val) on γSyn amyloid formation. These were M38/E110 (the most common human γSyn sequence), the SNP rs9864-driven M38/V110, alongside I38/E110, and the I38/V110 combination found in the two individuals described above (Fig. 2*A*). Initial assays in which Strep-tagged proteins were incubated for 13 d at 37 °C, pH 8.0 with shaking, showed that both proteins containing Ile38 were visibly more turbid than the M38/E110 and M38/V110 variants (although increased turbidity was also observed for M38/V110 compared with M38/E110) (Fig. 2*B*). Next, the kinetics of amyloid formation of the four untagged proteins was monitored using continuous ThT assays at four pH values (pH 4.5, 5.5, 6.5, and 7.5) (Fig. 2*C*). These pH values were chosen to mimic lysosomal, endosomal, and cytosolic pH, known to be important in αSyn aggregation into amyloid (52–54), and building on previous studies which have shown that αSyn amyloid formation occurs more rapidly at acidic pH (54–56). In all experiments, a total ionic strength of 20 mM was employed. The rate of amyloid formation of αSyn under these conditions was also measured for comparison, and the morphology of the resulting aggregates was visualized using negative stain EM (Fig. 2*D*). The results showed, as expected (9–11), that αSyn formed amyloid fibrils more rapidly than γSyn M38/E110 and M38/V110 under all conditions studied, with fibril formation proceeding most rapidly at lower pH values (Fig. 2*C*) (lag times and t_50_ values are reported in *SI Appendix*, Table S1). Notably, few, if any, fibrils resulted when γSyn M38/E110 was incubated at pH 7.5, in marked contrast with the behavior of αSyn which rapidly forms fibrils at this pH. γSyn M38/V110 formed amyloid even more slowly than M38/E110 at all pH values studied, with no fibrils forming at pH 6.5 and 7.5, and slow assembly occurring at the lower pH values. Thus, the substitution E110V, which removes a single negative charge from the C-terminal region of the protein, has a “protective” effect by slowing amyloid formation. This contrasts with the known effect of deleting negatively charged residues from the C terminus of αSyn (including deletions from D119) which accelerates fibril formation (57).

Strikingly, both γSyn variants containing the M38I substitution (I38/E110 and I38/V110) formed fibrils at all four pH values studied and resulted in rapid fibril assembly relative to their M38/E110 and M38/V110 counterparts (Fig. 2 *C* and *D* and *SI Appendix*, Table S1). Again, the inclusion of the substitution E110V slows fibril formation, with I38/V110 forming fibrils more slowly than I38/E110 at all pH values. The results portray the importance of residue 38 in the P1 region in controlling the rate of amyloid formation of γSyn and demonstrate that the single residue substitution of Met to Ile in this region dramatically enhances the rate of amyloid formation [note that γSyn I38/E110 and αSyn form amyloid at similar rates at pH ≤ 6.5 (*SI Appendix*, Table S1)]. This feat is even more remarkable given that the γSyn-NAC peptide did not form amyloid fibrils in isolation at pH 7.5 (compare Figs. 1*D* and 2*C*).

The relationship between pH and γSyn amyloid fibril assembly is complex (*SI Appendix*, Fig. S2). αSyn rapidly forms fibrils at all pH values studied, albeit at a slower rate than the αSyn-NAC peptide alone, consistent with the known role of the regions flanking NAC in protecting αSyn from self-assembly (41, 42). By contrast, γSyn I38/E110 and I38/V110 have accelerated fibril assembly at pH ≥ 6.5 relative to γSyn-NAC but assemble into amyloid more slowly than γSyn NAC at pH ≤ 5.5. The wider pH range over which γSyn I38/E110 forms amyloid relative to γSyn-NAC shows that the P1 region plays a greater role than simply modulating NAC sequestration during γSyn aggregation.

Analysis of the aggregate yield monitored by ultracentrifugation (Fig. 2*E* and *SI Appendix*, Fig. S3) confirmed the formation of fibrils for αSyn and γSyn I38/E110 under all conditions, with ≥87% of αSyn and ≥67% of γSyn I38/E110 forming pelletable (i.e., large) aggregates at the end of the incubation at all four pH values studied (Fig. 2*E* and *SI Appendix*, Table S1). By contrast, for both M38-containing γSyn variants, ≤27 and ≤15% of the protein was found in the pellet even at the lowest pH value studied (Fig. 2*E*), with fibrils typical of amyloid detected only for M38/V110 at pH 4.5 and 5.5 (Fig. 2*D*). After a brief sonication (*SI Appendix*, *Methods*), samples were reincubated for a further 48 h to determine whether assembly-competent protein remained in the sample. The results showed no further change in ThT signal, or yield of insoluble material subsequent to the additional sonication step (*SI Appendix*, Fig. S4), consistent with the values from the pelleting assays being representative of the critical concentration for fibril formation for these variants.

Finally, to confirm that the most aggregation-prone γSyn variant, I38/E110, forms a cross-β structure typical of amyloid, fibrils formed at pH 6.5 were analyzed using cryoEM. The results showed the formation of long straight fibrils that lacked an obvious twist (*SI Appendix*, Fig. S5). While the lack of a helical twist ruled out determination of the fibril structure to atomic resolution, analysis of 2D class averages and the periodicity of intensity along the fibril axis confirmed the formation of fibrils with a 4.8 Å repeat canonical of amyloid (58).

### Soluble Oligomers Are Enriched in γSyn I38 Variants.

The presence of isoleucine at position 38 of γSyn enhances amyloid formation at all pH values tested, but at pH 7.5, *ca.* 30% of I38/E110 protein was found not to pellet at 100,000*g* (the “soluble” fraction) (Fig. 2*E*). By contrast, for the M38-containing variants >75% of protein remained in the soluble fraction at the end point of the ThT assays (Fig. 2*E*). To assess the species that were present at the end point of the ThT reactions in more detail, each variant was subjected to rate-zonal density-gradient ultracentrifugation on a discontinuous sucrose gradient which has previously been used to separate αSyn monomers, oligomers, and fibrils (2).

**Fig. 3.**
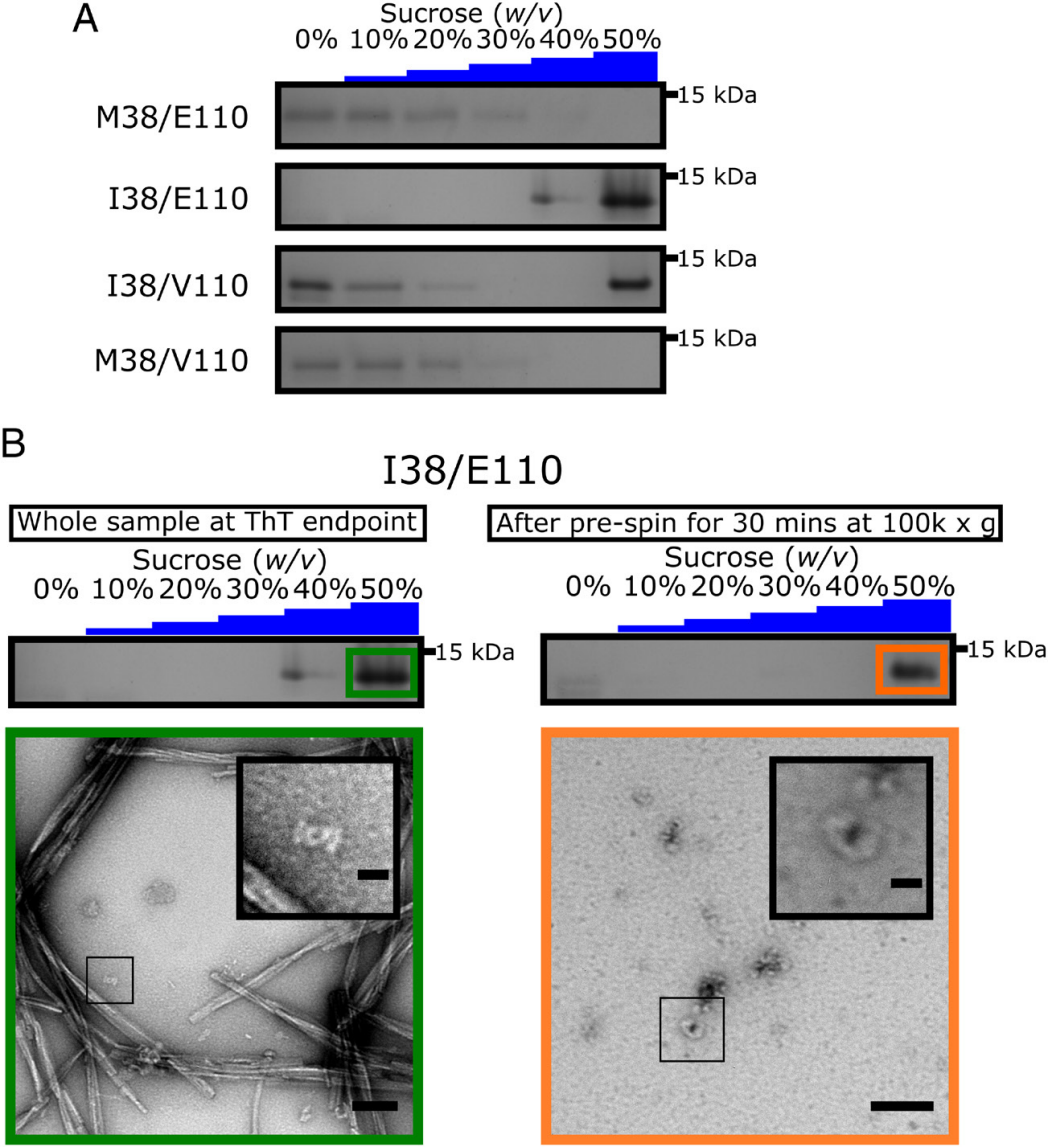
γSyn I38 forms distinct oligomeric species. (*A*) SDS-PAGE analysis of the γSyn variants at pH 7.5 after zonal density gradient ultracentrifugation at 113,000*g* for 4 h in a discontinuous sucrose gradient. The entire gels are shown in *SI Appendix*, Fig. S7 *A*–*D*. (*B*) (*Top*) SDS-PAGE showing that the γSyn I38/E110 species which do not pellet at 100,000*g* after 30 min in buffer are found exclusively in the 50% (*w/v*) sucrose fraction following zonal density gradient ultracentrifugation. The entire gel is shown in *SI Appendix*, Fig. S7*E*. (*Bottom*) Negative stain TEM micrographs representing the 50% (*w/v*) sucrose fraction of γSyn I38/E110 before (*Left*, Scale bar, 100 nm) and after removal of fibrillar material (*Right*, Scale bar, 50 nm). A zoom of each sample is shown *Inset.* (Scale bars, 20 nm.)

Control experiments, using freshly dissolved γSyn (M38/E110) monomers showed that the protein is found in fractions containing ≤10% (*w/v*) sucrose, whereas preformed αSyn fibrils were found exclusively in the 50% (*w/v*) sucrose layer (*SI Appendix*, Fig. S6). Analysis of the M38/E110 and M38/V110 variants at the end point of the ThT assays showed that the protein is mostly found in fractions containing ≤10% (*w/v*) sucrose, with small amounts of protein also found in the 20 to 30% (*w/v*) sucrose layers and none in the 50% (*w/v*) sucrose fraction, consistent with the majority of the protein remaining monomeric or forming small amounts of low density oligomers at the reaction end point (Fig. 3*A* and *SI* *Appendix*, Fig. S7 *A* and *B*). By contrast, the I38/E110 variant was found in the 40% and 50% (*w/v*) sucrose layers (Fig. 3*A* and *SI Appendix*, Fig. S7*C*), consistent with the formation of fibrils in high yield, as visualized by negative stain EM (Fig. 2 *D* and *E*). For the I38/V110 variant, monomers (≤10% (*w/v*) sucrose fractions), high-molecular-weight aggregates/fibrils (50% (*w/v*) sucrose fractions), and low-density oligomers (20% (*w/v*) sucrose fractions) coexist at the end of the incubation (Fig. 3*A* and *SI Appendix*, Fig. S7*D*), consistent with V110 slowing aggregation into amyloid as visualized by ThT fluorescence (Fig. 2*C*).

Finally, to separate fibrils from dense oligomers that could cosediment in the 50% (*w/v*) sucrose fraction of I38/E110, samples were first subjected to differential pelleting at 100,000*g* for 30 min which is sufficient to pellet all fibrillar material (59), and the supernatant was then immediately subjected to rate-zonal density-gradient ultracentrifugation to examine the nature of the species that remain. The I38/E110 protein was found exclusively in the 50% (*w/v*) sucrose fraction (Fig. 3*B* and *SI Appendix*, Fig. S7*E*). Negative stain TEM images of these samples confirmed that they contained oligomers (Fig. 3*B*), demonstrating that the Ile38 substitution enhances both oligomer and fibril formation.

### Lipid-Mediated Amyloid Formation Is Enhanced for γSyn I38/E110.

Lipid-mediated amyloid formation has been shown to be important in αSyn amyloid formation, and the effect of several disease-relevant αSyn variants can only be rationalised in the context of lipid membranes (60, 61). Accordingly, the ability of the γSyn variants to form amyloid fibrils, in quiescent conditions without added beads and instead catalyzed by liposomes [100 nm diameter, created using 1,2-dimyristoyl-sn-glycero-3-phospho-L-serine (DMPS)] was assessed using ThT fluorescence and negative stain EM, according to well-established protocols developed using αSyn (62). Strikingly, in the presence of DMPS liposomes at 0, 4, 8, 16, and 60:1 lipid to protein ratio (LPR), only I38/E110 was able to assemble into amyloid fibrils at pH 6.5 within 150 h at an initial monomer concentration of 50 μM (*SI Appendix*, Fig. S8 *A* and *B*). Interestingly, fibril formation was observed at all lipid concentrations used for this variant, although lipid-stimulated fibril assembly was greatest at an LPR of 16:1.

To determine whether all of the ySyn variants are able to bind to the DMPS liposome surface, despite fibril assembly not occurring for three of the four variants studied, each protein (25 μM monomer) was mixed with increasing concentrations of liposomes and binding (indicated by helix formation, monitored using far UV CD) was assessed (*SI Appendix*, Fig. S8*C*). All variants formed α-helix upon incubation with liposomes. The M38 variants were found to bind to the liposomes with a K_d_ of 4.6 ± 1.4 μM for M38/E110 and 7.6 ± 5 μM for M38/V110 [with L values (the number of lipid molecules bound per γSyn variant) of 29 ± 3 and 19 ± 7], which is similar to the range of values for these parameters reported for αSyn (41, 62, 63) (*SI Appendix*, Fig. S8*D*). Notably, however, binding of the M38 proteins resulted in comparatively little helix formation (~13 and 9%), compared with 64% for αSyn (41) (*SI Appendix*, Fig. S8*C*). This behavior is reminiscent of that previously reported for the αSyn variant ΔΔ, which lacks residues 36–56, and in which relatively little helix is again formed upon lipid binding, while the K_d_ is unperturbed (41). In this case, however, fibril formation does result from liposome binding (41). In contrast with the behavior of the M38 γSyn variants, the I38 variants bind DMPS liposomes less tightly, with binding remaining unsaturated (and the K_d_ unfittable) even at the highest LPR studied. However, even though binding more weakly, the percent helix formed of the I38 γSyn variants at high LPRs is larger (34 and 26% at the highest LPR measured) with fibrillar structures resulting only from I38/E110 (*SI Appendix*, Fig. S8 *B* and *C*). This demonstrates that even with weaker binding, greater helix results for the I38-containing γSyn variants. This is unexpected as there is little difference in the helical propensity of Met and Ile. I38/E110, therefore, promotes fibril assembly in the presence of liposomes, despite binding more weakly to them.

### γSyn I38 Aggregation Is Enhanced in Cells but Does Not Result in a Toxic Phenotype in *Drosophila* Models.

**Fig. 4.**
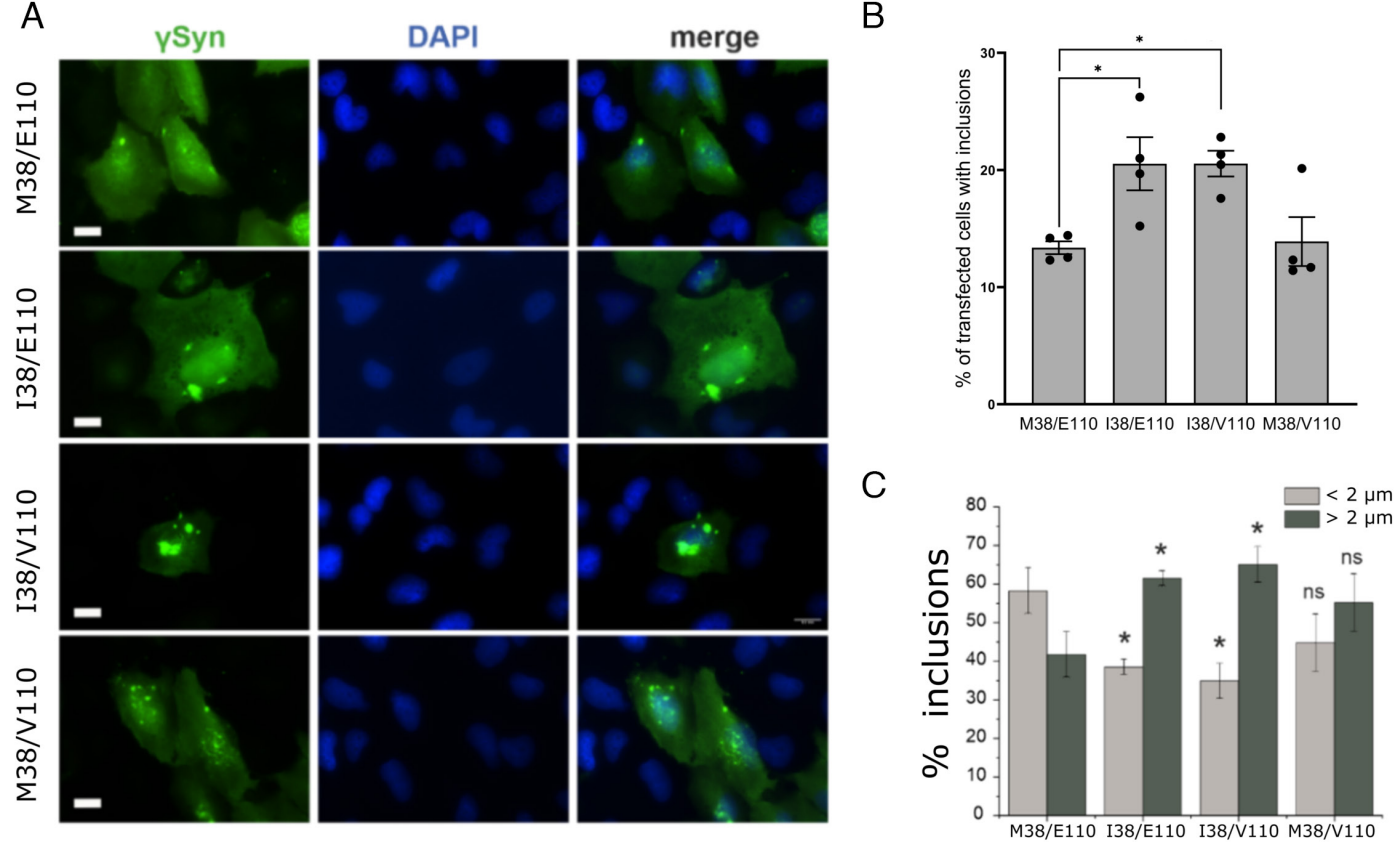
Inclusions formed by γSyn variants in human H4 cells. (*A*) Fluorescence microscope images of cultured cells 48 h after transfection with the respective expression plasmids. The cultured cells were immunostained with anti-γSyn antibody and nuclei counterstained with DAPI. (Scale bar, 20 µm.) (*B*) Quantification reveals an increase in the number of transfected cells that formed inclusions after expression of γSyn I38/E110 and I38/V110 variants, compared to expression of M38/E110 and M38/V110 variants. The bar chart shows mean ± SEM of percentage of successfully transfected cells bearing inclusions (*P < 0.05, one-way ANOVA, *n* = 4 independent experiments with >100 cells assessed in each experiment). (*C*) Analysis of inclusion size reveals that cells expressing γSyn I38/E110 or I38/V110 variants form larger inclusions more frequently than cells expressing the M38/E110 variant. The bar chart shows mean ± SD of percent of small (diameter <2 µm, light gray bars) and large (diameter >2 µm, dark gray bars) aggregates in transfected cells (*P < 0.05, Student's *t* test for independent variables).

To examine the effect of amino acid substitutions at residue 38 of γSyn in a cellular context, each of four γSyn variants was transiently expressed in human H4 neuroglioma cell lines, a cellular model that has been used extensively to assess the formation of αSyn inclusions (64, 65). Consistent with the in vitro data presented above, cells expressing the γSyn I38/E110 or I38/V110 variants were more likely to form γSyn inclusions (visualized with an anti-γSyn antibody) than cells expressing either the M38/E110 or M38/V110 variants (Fig. 4 *A* and *B*). Analysis of inclusion size revealed a significant increase in the number of large inclusions (>2 µm in diameter) and a decrease in the number of small inclusions (<2 µm in diameter) in cells expressing I38/E110 and I38/V110 compared with cells expressing the equivalent M38 variant, with no evidence of cell death associated with aggregate formation, as judged by these experiments (Fig. 4*C*).

Previous studies demonstrated that neuronal expression of human αSyn, particularly variants with increased propensity to aggregate into amyloid (e.g., A53T), can cause changes in certain aspects of the transgenic *Drosophila* phenotype, making this organism a useful model of pathological processes associated with PD and other α-synucleinopathies (66, 67). To elucidate whether expression of the γSyn M38 or I38 variants causes an effect in an organismal context, we produced transgenic *Drosophila* lines expressing the four γSyn variants in the fly eyes, pan-neuronally or in glutamatergic neurons (*SI Appendix*, *Methods*). Similar levels of expression of the γSyn variants were detected in the four transgenic lines by western blotting (*SI Appendix*, Fig. S9*A*), but we found no evidence of degeneration in the eyes or evidence for the formation of large aggregates in glutamatergic neurones (*SI Appendix*, Fig. S9 *B* and *C*). Although three of the four variants showed a significantly decreased ability in the RING climbing assay in aged flies compared with controls (*SI Appendix*, Fig. S9*D*), lifespan was not compromised (in fact, with the exception of I38/V110, lifespan was marginally increased) (*SI Appendix*, Fig. S9*E*). The results highlight the importance of the biological context in assessing aggregation and proteotoxicity, which can depend on cell type, expression level, and other cellular effects (e.g., chaperone machinery, degradation rates) that may mitigate γSyn aggregation and its associated potential toxicity in different models (68–70). Previous experiments have shown that binding of γSyn to phospholipase *Cβ2* or to liposomes formed of POPC/POPS, disfavours oligomer formation in vitro and in cells (71). Further experiments at the whole proteome level will be needed to understand the biological mechanisms that prevent the accumulation of γSyn aggregates in these fly models.

### Amyloid Assembly of γSyn Depends on the Identity of the Residue at Position 38.

**Fig. 5.**
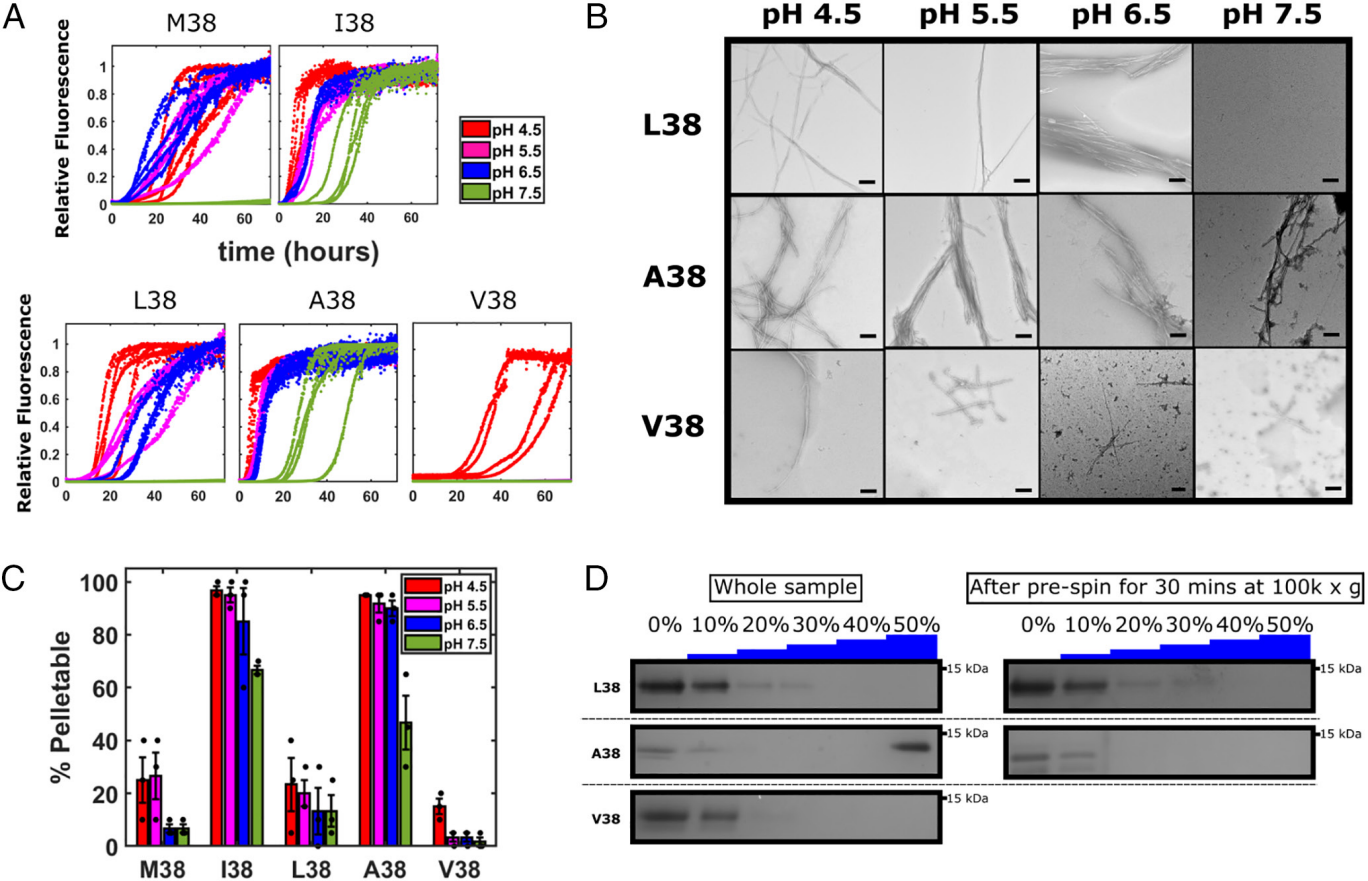
Amyloid fibril assembly by γSyn is controlled by the identity of the residue at position 38. (*A*) ThT fluorescence of γSyn variants with different substitutions at position 38 monitored at 37 °C in Tris/sodium acetate buffers at 20 mM ionic strength at pH 4.5 (red), 5.5 (pink), 6.5 (blue), and 7.5 (green). The M38/E110 and I38/E110 data from Fig. 2 are included here for comparison. Four replicates are shown. Lag time and T_50_ values are shown in *SI Appendix*, Table S1. (*B*) Negative stain TEM images of the end point samples of the ThT reactions. (*C*) Differential pelleting assay demonstrating the relative amount of protein that was found in the pellet in samples taken at the end point of the ThT assays after centrifugation at 100,000*g* for 30 min. The mean ± SD is shown (see also *SI Appendix*, Table S1). (*D*) SDS-PAGE analysis of samples generated at pH 7.5 analyzed by rate-zonal density-gradient ultracentrifugation before and after the differential pelleting assay. All variants in these experiments also have E110.

We have shown previously that the side chain at position 38 in αSyn is important in determining the ability of the protein to form amyloid, with L38M inhibiting, L38I enhancing the rate of amyloid formation, and L38A behaving similarly to the parent Leu38 (42). To determine whether other aliphatic side chains at position 38 can enhance γSyn amyloid formation, residue 38 in γSyn was substituted with Leu, Val, or Ala (each contained E110), and the effect of these substitutions on the rate and yield of amyloid formation was determined, with differential centrifugation being used to characterize oligomers formed (*SI Appendix*, Fig. S10). Remarkably, and by contrast with I38, the substitution L38 resulted in the formation of fewer, if any, fibrils at pH 7.5, slower fibril formation compared with I38 at more acidic pH, and a lower (<30%) pellet yield (Fig. 5 *A*–*C* and *SI Appendix*, Table S1), consistent with our previous results on this variant (42). The substitution V38 slowed assembly compared with Met at this position at all pH values tested, while A38 increased the rate of amyloid assembly compared with M38 at all pH values, with fibrils resulting for the latter protein even at pH 7.5. The T_50_ values (across all measurable conditions) thus follow the rank I38 ~ A38 << L38 < M38 < V38 (*SI Appendix*, Table S1). The percent pelletable material follows the same rank order and is dependent on the identity of residue 38 (Fig. 5*C*). Oligomers formed by the different variants at pH 7.5, identified using rate-zonal density-gradient ultracentrifugation, as described above, yielded results that mirror the ThT and pelleting assay results, wherein M38 and V38 sediment mostly in the 0 to 20% (*w/v*) fractions consistent with monomers/small oligomers (Figs. 3*A* and 5*D*), L38 forms a mixture of monomers/small oligomers that sediment in 20 to 30% (*w/v*) sucrose, and A38 partially remains monomeric and partially converts to fibrils that pellet in 50% (*w/v*) sucrose, while I38 forms fibrils and oligomers at pH 7.5 that each pellet in 50% (*w/v*) sucrose (Fig. 3*B* and 5*D* and *SI Appendix*, Fig. S11).

While the mechanism(s) behind the different outcomes of assembly of these conservative substitutions remain unclear [neither the rank order of T_50_ values at pH 4.5 or pellet yield at pH 7.5 correlate with solubility (Camsol (30)), amyloidogenicity (AmylPred (72)), β-sheet propensity (73), or helicity (AGADIR (74)), *SI Appendix*, Fig. S12], the data demonstrate that conservative changes of single residues in the P1 region of γSyn can tip the balance toward or away from amyloid formation, despite 126 of its 127 residues remaining unchanged.

## Discussion

Despite decades of research, how and why proteins self-assemble into amyloid fibrils associated with pathogenic human diseases remains unclear, and why different cells and tissues have different susceptibility to oligomers and fibril formation remains mysterious (75). Algorithms have been developed, trained using data obtained on peptides and proteins, which enable the identification of short sequences with high aggregation propensity, known as aggregation-prone regions (APRs) (76–78). Such regions are commonly required for amyloid formation of the proteins that contain them, with the APR frequently forming part of the structured core of the resulting fibrils (79, 80). For natively folded proteins, APRs tend to be sequestered within their 3D structure, protecting the proteins from aggregation, and simultaneously playing a key role in stabilizing their hydrophobic cores (79, 80). This rationalizes why partial, or complete, unfolding of natively structured proteins is a necessary precursor of amyloid formation, as shown for antibody light chains (81, 82), transthyretin (83, 84), and β_2_-microglobulin (85, 86), in which their amyloid structure is unrelated to their native folds.

APRs are also found in IDPs associated with amyloid disease, such as Aβ, islet-associated polypeptide (IAPP) and αSyn involved in Alzheimer’s, type II diabetes and PD, respectively. However, even though these APRs are not encumbered by a folded structure, it is still difficult to predict the rates of assembly into amyloid of the proteins that contain these APRs, or the effects of the solution conditions (56), sequence changes (41, 42, 87), or posttranslational modifications (88, 89) on amyloid formation. This arises since the propensity to form amyloid depends on a complex interplay between different properties of the proteins, including their solubility and secondary structure propensity (49), the number and strength of their APRs (77), how the regions that flank an APR modulate its amyloid potential (90), the nature of early protein–protein interactions (91), the formation of trapped off-pathway oligomers (92, 93), and the structure and stability of the resulting amyloid fibrils themselves (94, 95). In a cellular context, binding to chaperones (96), other proteins (97), membranes (98), or other biomolecules (99) can tailor amyloid formation, favoring or disfavouring fibril assembly dependent on the precise matching of the IDP sequence with the cellular environment. This raises important questions about how IDPs self-assemble into amyloid and gain order, and how this self-assembly process yields different cross-β fibril structures associated with different diseases from similar, or identical, precursor sequences (24, 25).

The self-assembly of αSyn into amyloid is a particularly perplexing example of amyloid formation, wherein the aggregation-prone, central NAC region has been shown to be necessary and sufficient for amyloid formation (21–23), yet familial mutations associated with PD lie outside NAC. This finding is consistent with the notion that the regions which flank an APR can have dramatic effects on assembly (42, 86). While some familial mutations accelerate αSyn aggregation into amyloid in vitro [e.g., A53T (100) and A53V (101)], others slow assembly [e.g., A30P (11)], and some have relatively little effect [e.g., A30G (33) and A53E (101)], with the rank order of the effects of the familial variants on amyloid formation depending on the conditions used. αSyn molecules containing truncations in the C-terminal region, from residues 124, 119 and 102 which are enriched in Lewy body deposits (102) have been shown to accelerate amyloid formation (46), while deletion of the P1 and P2 regions that lie N-terminal to NAC slow or ablate amyloid formation in vitro and in *C. elegans* models (41, 42). These findings highlight the crucial role of the regions that flank NAC in determining αSyn amyloidogenicity in vitro, in cells, and in animal models.

Despite being implicated in neuronal pathology associated with neurodegenerative diseases, including ALS (17–19), the aggregation of γSyn into amyloid has not been studied widely. The few studies reported show that γSyn does not form amyloid in vitro without resorting to low pH (e.g., pH 3) or protein concentrations exceeding 250 μM, which concur with the finding that γSyn inhibits amyloid formation of αSyn (9–11). Here, inspired by our identification of a rare polymorphism in the *SNCG* gene of two individuals with ALS in the absence of mutations in the SOD1, TDP-43, C9orf72, FUS, and ANG genes commonly associated with ALS, we explored the role of the identified sequence change (Met38 for Ile), which lies within in the P1 region of γSyn, in modulating its ability to form amyloid fibrils in vitro, in cells and in fly models. Remarkably, we show that while γSyn, and its NAC peptide, do not form amyloid fibrils in vitro at pH 7.5, at least under the conditions explored, the replacement of Met38 with Ile in the P1 region results in rapid and quantitative formation of fibrillar assemblies with an ordered, 4.8 Å repeat canonical of an amyloid fold. I38/E110 was also the only variant of γSyn of the four proteins studied which formed amyloid fibrils upon incubation with liposomes. Substitution of residue 38 with other aliphatic amino acids demonstrated a remarkable sensitivity of the protein behavior to the identity of the side chain at this position, with Leu having little effect, Ala promoting assembly, and Val disfavoring amyloid formation relative to Met. The individuals we identified with Ile38 in γSyn also contained Val at position 110, the latter corresponds to a known SNP in the *SNCG* gene in the human population (50). We show that the substitution of Glu for Val at position 110 retards the rate of amyloid formation, irrespective of the identity (Met or Ile) of residue 38, or whether the fibril assembly reaction was stimulated by DMPS liposomes.

While overexpression of the four γSyn variants studied here in a cell model resulted in the formation of in-cell inclusions, which were enhanced in number and size in the I38-containing γSyn variants, a toxic phenotype was not observed in the cells, nor was toxicity observed when these proteins were expressed pan-neuronally, in the eyes, or in glutamergic neurones in *Drosophila* models. Why this is the case is not clear, but a similar observation (an increased lifespan with no deleterious phenotype) has been reported for the expression of superoxide dismutase in *Drosophila* (103). Further work will be needed to unpick the cellular responses that could modulate or reduce the amyloid- and proteotoxic potential of γSyn in these models (70). Because it is unknown whether amyloid-like aggregates are observed in the nervous system of the ALS patients carrying the M38I γSyn substitution and the family history of the disease for these patients is unavailable, a direct link between high propensity of M38I γSyn to aggregate in vitro and in cultured cells and pathology associated with ALS cannot be made. Posttranslational modifications of γSyn which were not considered in our study, could ameliorate (or exacerbate) amyloid formation and its associated proteotoxicity in these cases. Notably, oxidation of Met38 in γSyn has been found, colocalized with αSyn, in cytoplasmic inclusions in neurones in the amygdala and substantia nigra of individuals with DLB, suggestive of a role of residue 38 in γSyn aggregation in a clinical setting (48). It is also worth noting that the I38 variants of γSyn both demonstrated weaker binding to liposomes than the M38 proteins, an effect that was not mitigated by V110.

The results presented highlight the importance of residue 38 in the P1 region of γSyn in defining the rate of amyloid formation, akin to the behavior of αSyn as shown in our previous studies (41, 42). This suggests that the two proteins share similarities in their mechanisms of amyloid assembly and provide a second, striking example of the critical importance of the P1 region in controlling amyloid formation. In contrast to our previous findings for αSyn which suggest that the flanking regions modulate amyloid propensity by directly or indirectly affecting the accessibility of NAC (41, 42), the data presented here suggest that P1 may enable an alternative aggregation pathway in γSyn to that driven by NAC. This is suggested by the decreased aggregation propensity of γSyn-NAC relative to αSyn-NAC, and the finding that the γSyn I38 and A38 variants form amyloid more rapidly than the isolated NAC peptide at pH ≥ 6.5. A switch in mechanism would also be consistent with the nonlinear dependence of lag time on pH (*SI Appendix*, Fig. S2). Interactions between NAC and the C-terminal flanking region must also be involved in the mechanism of aggregation of γSyn into amyloid, since switching residue 110 from Glu to Val retards amyloid formation irrespective of the identity of residue 38. Further work will be needed to understand precisely how these side chains modulate assembly.

Regardless of the mechanism(s) by which the Met to Ile switch at residue 38 results in enhanced amyloid formation of γSyn in vitro, the remarkable sensitivity of the amyloid potential of the protein to the identity of the residue at position 38, as well as the role of residue 110 in retarding amyloid formation, highlights how the protein behavior is balanced on a knife’s edge. Our finding that amyloid formation is controlled by the sequence of the P1 region in both αSyn and γSyn suggests that the different diseases associated with their aggregation could share common mechanistic features. Further comparative studies of the two proteins will thus provide a powerful platform to better understand how and why these IDPs aggregate into amyloid.

## Materials and Methods

Details of all protocols are provided in *SI Appendix*, *Methods*.

### ThT Fluorescence Assays.

Buffers were prepared by mixing Tris and sodium acetate to give Tris/sodium acetate buffers at pH 4.5, pH 5.5, pH 6.5, and pH 7.5 each with a total 20 mM ionic strength. De novo amyloid assembly assays were performed by incubating 80 µM synuclein (αSyn/γSyn) or NAC peptide in buffer containing 20 μM ThT at 37 °C with a single Teflon coated poly-ball per well and continuous shaking at 600 rpm. Where necessary tip sonication was performed to create fibril seeds and in some experiments the sample was incubated and monitored for a further 48 h after sonication. The products of assembly were monitored using negative stain EM and by pelleting assays on a sucrose gradient.

### Mutagenesis, Expression, and Purification.

γSyn variants with single amino acid substitutions were generated by Q5 site directed mutagenesis and the protein expressed and purified as described previously (41).

### Genomic Analysis.

Genomic DNA was extracted from blood leukocytes and the whole coding region of the *SNCG* gene analyzed by direct sequencing.

### CryoEM Sample Preparation and Analysis.

γSyn-I38/E110 fibrils were formed at pH 6.5 from 100 μM monomer in 1.5 mL Eppendorf tubes at 37 °C with shaking at 600 rpm, followed by two weeks incubating quiescently at room temperature. The cryoEM dataset was collected at the University of Leeds Astbury Biostructure Laboratory using a Titan Krios electron microscope (Thermo Fisher). Fibrils from roughly 100 micrographs were manually picked and the data were processed using RELION-4.

### Fibril Formation in the Presence of Liposomes.

Amyloid formation in the presence of DPMS liposomes was monitored using ThT fluorescence under quiescent condition using 50 μM initial monomer, 30 °C, with no beads in 20 mM sodium phosphate buffer, pH 6.5. Binding to liposomes was monitored using far UV CD as described in ref. 41.

### Cell Culture.

The four γSyn variants were transiently expressed in human neuroglioma cells (H4). After 48 h cells were fixed and aggregates of each variant probed by immunostaining as described in *SI Appendix*, *Methods*.

## Supplementary Material

Appendix 01 (PDF)

## Data Availability

The data are available in the University of Leeds Data depository (https://doi.org/10.5518/1315) (104).
