# Supplementary material for: Substitution of Met-38 to Ile in γ-synuclein found in two patients with amyotrophic lateral sclerosis induces aggregation into amyloid
Source: Proc Natl Acad Sci U S A. 2024 Jan 3;121(2):e2309700120. doi: 10.1073/pnas.2309700120 (PMC10786281; doi:10.1073/pnas.2309700120)
Supplement: Supplementary file 1 — Appendix 01 (PDF) [file pnas.2309700120.sapp.pdf]

Supplementary information for

"Substitution of Met-38 to Ile in γ-synuclein found in two patients with amyotrophic lateral sclerosis induces aggregation into amyloid"

Liam D Aubrey[a1], Natalia Ninkina[b,c1], Sabine M. Ulamec[a], Natalia Y. Abramycheva[d], Eftychia Vasili[e], Oliver M. Devine[a], Martin Wilkinson[a], Eilish Mackinnon[b], Galina Limorenko[b,e], Martin Walko[a,f], Sarah Muwanga[b], Leonardo Amadio[b], Owen M. Peters[b], Sergey N. Illarioshkin[d], Tiago F. Outeiro[g,h,i,j], Neil A Ranson[a,], David J. Brockwell[a,2], Vladimir L. Buchman[b,c,2], Sheena E. Radford[a,2]

[a]Astbury Centre for Structural Molecular Biology, School of Molecular and Cellular Biology, Faculty of Biological Science, University of Leeds, Leeds LS2 9JT, United Kingdom

[b]School of Biosciences, Cardiff University, Cardiff CF10 3AX, United Kingdom

[c]Department of Pharmacology and Clinical Pharmacology, Belgorod State National Research University, Belgorod 308015, Russian Federation

[d]Research Center of Neurology, Moscow 125367, Russian FederationLaboratory of Neurobiology and Tissue Engineering, Brain Science Institute, Research Center of Neurology,Moscow 125367, Russia

[e]Laboratory of Molecular and Chemical Biology of Neurodegeneration, Institute of Bioengineering, School of Life Sciences, Ecole Polytechnique Fédérale de Lausanne, CH-1015 Lausanne, Switzerland

[f]Astbury Centre for Structural Molecular Biology, School of Chemistry, University of Leeds, Leeds LS2 9JT, United Kingdom

[g]Department of Experimental Neurodegeneration, Center for Biostructural Imaging of Neurodegeneration, University Medical Center Göttingen, 37075 Göttingen, Germany

[h]Max Planck Institute for Multidisciplinary Sciences, 37075 Goettingen, Germany

[i]Translational and Clinical Research Institute, Faculty of Medical Sciences, Newcastle University, Newcastle Upon Tyne NE2 4HH, United Kingdom

[j]Scientific employee with a honorary contract at Deutsches Zentrum für Neurodegenerative Erkrankungen, Göttingen37075 Göttingen, Germany

Corresponding authors:

Sheena E Radford
Tel: +113 343 3170, Email: S.E.Radford@leeds.ac.uk

David J Brockwell
Tel: +113 343 7821; Email: D.J.Brockwell@leeds.ac.uk

Vladimir L. Buchman
Tel: +442920879068; Email: buchmanvl@cf.ac.uk

Author Contributions: L.D.A., S.M.U., M.W., S.N.I., T.F.O., N.A.R., D.J.B., V.L.B., and S.E.R. designed research; L.D.A., N.N., S.M.U., N.Y.A., E.V., O.M.D., M. Wilkinson, E.M., G.L., M. Walko, S.M., L.A., O.M.P., and S.N.I. performed research; L.D.A., N.N., S.M.U., O.M.D., M. Wilkinson, E.M., G.L., M. Walko, S.M., L.A., O.M.P., S.N.I., T.F.O., N.A.R., D.J.B., and V.L.B. analyzed data; and L.D.A., N.N., S.M.U., N.Y.A., E.V., O.M.D., M. Wilkinson, G.L., M. Walko, L.A., O.M.P., S.N.I., T.F.O., N.A.R., D.J.B., V.L.B., and S.E.R. wrote the paper.

[1] L.D.A. and N.N. contributed equally to this work.

This PDF file includes:

Materials and Methods
Supplementary Text
Supplementary Figures 1 to 12
Supplementary Table 1

Materials and methods

In silico analysis

The αSyn and γSyn sequences were aligned using the UniProt align tool available at www.uniprot.org/align (1). The aggregation propensity and solubility of the sequences were analysed by using the online tools Zyggregator (2) and CamSol (3) at pH 7.0 or 4.0.

NAC peptide synthesis

Peptides (NH_2-EQVTNVGGAVVTGVTAVAQKTVEGAGSIAAATGFV-COOH and NH_2-EQANAVSEAVVSSVNTVATKTVEEAENIAVTSGVV-COOH for αSyn-NAC and γSyn-NAC, respectively) were synthesised on a CEM Liberty Blue peptide synthesiser with microwave assistance using default coupling cycles for the first 15 residues and double couplings for the rest of the peptide. The synthesis was performed on 0.1 mmol scale using Valine preloaded HMPB-NovaPeg resin (0.56 mmol/g), DMF as a solvent, 20% (*v/v*) piperidine in DMF for the deprotection and 5 eq. of Fmoc-protected amino acid, 5 eq. DIC and 5 eq. Oxyma for couplings. Cleavage from resin was accomplished using TFA:H_2O:TIS:EDT, 92.5 : 2.5 : 2.5 : 2.5 (5 mL x 3 h) and peptides were precipitated using cold ether. Peptides were purified on preparative HPLC Agilent 1260 infinity system equipped with UV detector, fraction collector and Kinetex EVO 5 µm C18 100Å 21.2 x 250 mm reverse phase column using a gradient of 10 - 30% (*v/v*) acetonitrile with 0.1% (v/v) ammonia, followed by lyophilisation. The identity of the peptides was confirmed by high-resolution mass spectrometry on Bruker Maxis Impact spectrometer using electrospray ionisation. Immediately before use the peptide was resuspended in the appropriate experimental buffer and an aliquot of this was diluted thousand-fold into water. The A_{205} of the diluted sample was measured using a dual beam UV-1800 spectrophotometer (Shimadzu) with extinction coefficients of 104,320 and 96,120 $M^{-1}cm^{-1}$ for αSyn-NAC and γSyn-NAC, respectively (4). The remaining undiluted peptide was diluted in reaction buffer to the appropriate concentration.

ThT fluorescence assays

Four ThT assay buffers were prepared by mixing Tris and sodium acetate, to give Tris/sodium acetate buffers at pH 4.5, pH 5.5, pH 6.5 and pH 7.5 each with a total 20 mM ionic strength.

De novo amyloid assembly assays were performed by incubating 80 µM synuclein protein (αSyn or γSyn) or NAC peptide solution in each assay buffer containing 20 µM ThT (each well containing a total of 100 µL solution) in sealed 96-well flat bottom assay plates (Corning, non-binding surface 3651) in a FLUOstar Omega plate reader (BMG Labtech) at 37°C with a single teflon coated poly-ball per well (PolyScience Inc, 17649-100) and continuous shaking at 600 rpm for 72 h. ThT fluorescence was exited at 444 nm, emission detected at 480 nm. Experiments, in general, were performed three times with four replicates. Data were processed in MATLAB (r2022b) where the raw data was normalised to the 95th percentile of the signal amplitude for samples that reached a plateau. Where appropriate, slopes in the plateau phase were removed by fitting the plateau phase data and subtracting the fitted values. The t_{lag} was calculated as the x-intercept of the tangent to the inflection point and the t_{50} as the time taken to reach y = 0.5 after normalisation. If a plateau was not reached within the experimental time frame or if an enhanced ThT signal was not observed at all then the signal was normalised to the 95th percentile of the average pH independent signal of the same variant in that microplate (all proteins showed a ThT positive signal at pH 4.5). Where necessary tip sonication was performed after 72 h using a 3 mm ultrasonic probe (Sonics, 630-0422) at 20 % amplitude with a VCX-130PB ultrasonic processor (Sonics) and the sample was redeposited into a fresh assay plate of the same type and incubated for a further 48 h whilst ThT signal was monitored.

Negative stain TEM

For negative stain TEM images, 5 µL of each sample was added to a glow discharged carbon coated copper grid and incubated for 20 sec. Samples were dried by dabbing with filter paper and the grid was then washed two times by submersion into a water droplet with drying steps in-between each wash. This process was repeated with two droplets of 1 % (*w/v*) uranyl-acetate to negatively stain the samples. Imaging was performed on either the FEI Tecnai T12 electron microscope or the FEI Tecnai F20 electron microscope. The NAC peptide samples were centrifuged and resuspended in 20 µl of water prior to deposition.

Genomic analysis

Genomic DNA was extracted from blood leukocytes using Wizard Genomic DNA Purification Kit (Promega). The whole coding region of the *SNCG* gene was analysed by direct sequencing. Nucleotide sequence analysis was performed on a capillary genetic analyser ABI

Prism 3130 (Applied Biosystems). Data Collection Software, (version v3.0), Sequencing Analysis Software (version v5.2) and SeqScape Software (version v2.5) were used for data analysis. The following primers flanking the *SNCG* exons with adjacent intronic regions (at least 50 nucleotide pairs from each end) were used: 5’-TGGAGGAAGGTGAGGCTGA-3’ and 5’-ACATAGGTGTGCACAGGGC-3' for exon I, 5’-CCCCCACATTCTGTCCTGTC-3’ and 5’-GCGCTCAGTGGGTACTGAAA -3' for exons II-III, 5’-TCATCAGAGCCCCGGGTATT-3’ and 5’-CCAGGTCCTCCCAGGAACA-3' for exons IV-V.

Mutagenesis, expression, and purification

γSyn variants with single amino acid substitutions were generated by Q5 site directed mutagenesis (NEB), starting with a plasmid containing human cDNA encoding M38/E110 variant of γSyn in a pET23a vector. Recombinant expression of the proteins was performed in *Escherichia coli* BL21 (DE3) cells and purification performed as described previously (5) before lyophilisation and storage at -20°C. Immediately before an experiment, the protein was dissolved in the desired buffer. The purity and correct mass of all proteins was confirmed by mass spectrometry (ESI-MS) and SDS-PAGE.

Strep-tagged γSyn variants and in-tube aggregation assay

A DNA fragment encoding Strep-tag was added in-frame just upstream the stop codon of each of four cDNAs encoding the human γSyn variants using a standard PCR technique and the resulting DNA fragments were cloned into the bacterial expression vector pCS19 (6). Expression of recombinant proteins in KU98 cells was induced by 0.5 mM IPTG for 5 h. Bacteria cells were then collected, lysed, and the cleared lysates incubated in a boiling water bath for 20 min. Denatured protein precipitates were separated from the γSyn-containing supernatant as described previously (7) and Strep-tagged γSyn variants were purified using Strep-tactin columns (IBA-GmbH, Goettingen, Germany) according to the manufacturer’s instructions. A solution of each protein (900 µL of 0.92 mg/mL protein) in diluted BXT elution buffer (final concentrations: 33 mM TrisHCl, 50 mM NaCl, 0.33 mM EDTA, 1.66 mM biotin, pH 8) was prepared in duplicate in sterile 2 mL test tubes and incubated at 37 ^{o}C with constant shaking at 190 rpm for 13 days.

Differential pelleting assay

Replicate samples from the endpoint of each ThT assay were pooled and 100 µL from each was loaded into a TLA100 ultracentrifuge tube (Beckman Coulter 343775) and subjected to ultracentrifugation at 100,000*g* for 30 min in a Beckman Coulter Optima MAX-XP Ultracentrifuge. The soluble fraction after ultracentrifugation was collected. Both the non-centrifuged (whole) and the soluble fraction were diluted 1 in 6 into the appropriate ThT assay buffer before being diluted further with SDS loading dye. The samples were depolymerised by boiling in 2% (*w/v*) SDS for 10 min and loaded onto 15% Tris-Tricine SDS-PAGE gels. The gels were stained using InstantBlue Coomassie protein stain (Abcam ISB1L) and imaged using an Alliance Q9 Imager (Uvitec). Band intensities were quantified using ImageJ version 1.52a. The percentage of pelleted protein was quantified from the intensity of the band corresponding to the whole fraction minus the intensity of the band corresponding to the soluble fraction.

Alternately for the NAC peptides, quantification was performed as above except that the soluble fraction was diluted 1 in 10 in 0.1% *(v/v)* trifluoroacetic acid (TFA) in a conical HPLC vial (Thermo Scientific C4010-13) and 10 µl was loaded onto a Nucleosil C4 300Å 250 x 4.6 mm HPLC column (Chromex) that had been equilibrated in 0.1% *(v/v)* TFA. Protein was eluted over 15 min on a gradient of 5-80% *(v/v)* acetonitrile using a Nexera LC-40 system (Shimadzu). Absorbance over a range of wavelengths from 190 nm to 350 nm was detected using a photodiode array detector (Shimadzu) and the absorbance at 205 nm was used in all calculations. 10, 7.5, 5, 2.5 and 1 µl of freshly dissolved 8 µM γSyn-NAC peptide (dissolved and initially diluted to 80 µM in water followed by dilution to 8 µM in 0.1% *(v/v)* TFA) was loaded onto and eluted from the same column, identically as before, to form a set of standards. The area under the curve (AUC) of the peak for each run at 9.5 min was calculated in MATLAB (r2022b) and the set of standards was fit to a straight line using the concentrations 8, 20, 40, 60 and 80 µM as the x axis coordinates. Finally, given that the original sample at the start of the ThT experiment was 80 µM, the concentration of the soluble protein was converted to percentage of sample in the pellet.

CryoEM sample preparation and data collection

γSyn-I38/E110 fibrils were formed at pH 6.5 from a 100 µM starting monomer concentration in 1.5 mL Eppendorf tubes with incubation for three weeks at 37˚C with shaking at 600 rpm, followed by two weeks incubating quiescently at room temperature. Prior to preparing cryo-EM grids, the fibrils were concentrated 4x-times by centrifuging 100 µL of the reaction at 13,000*g* for 10 min and resuspending the pellet in 25 µL of the resulting supernatant. The

sample was applied to Tergeo plasma cleaned (Pie Scientific, 60 s) Lacey carbon 300 mesh grids, blotted and frozen in liquid ethane using a Vitrobot Mark IV (FEI) with a 0 sec wait and 5.5 sec blot time, respectively. The Vitrobot chamber was maintained at close to 100% humidity and 6˚C. The cryoEM dataset was collected at the University of Leeds Astbury Centre Biostructure Laboratory using a Titan Krios electron microscope (Thermo Fisher) operated at 300 kV with a Falcon4 detector in counting mode and Selectris energy filter set to a 10 e-V slit width. A nominal magnification of 130,000x was used yielding a pixel size of 0.95 Å. A total of 5,280 movies were collected with a nominal defocus range of -1.3 to -2.5 µm and a total dose of ~41 e-/Å2 over an exposure of 5 s, corresponded to a dose rate of ~7.4 e-/pixel/s. The movies were collected as EER fractions with 1539 raw hardware frames.

CryoEM data processing

The raw EER movies were fractionated into 38 fractions, aligned and summed using motion correction in RELION-4 (8) with a dose per frame of 1.1 e-/Å2. CTF parameters were estimated for each micrograph using CTFFIND4 (9). Images that did not contain fibrils were removed by quickly screening through 10 Å lowpass-filtered micrographs paneled at a 0.2x scale, leaving 1,579 micrographs for further processing (SI Appendix Figure S5A). Fibrils from roughly 100 micrographs were manually picked, and fibril segments were extracted and used to train a picking model in crYOLO (10) to automatically pick from all the images with an inter-box spacing of 14.4 Å (three helical repeats). The resulting 584,854 segments were extracted 2x binned (with a 570 Å box size) and 483,631 segments remained after picking artefacts such as carbon edges and ice contamination were removed using multiple rounds of 2D classification using the VDAM algorithm in RELION-4 (SI Appendix Figure S5B).

There was no evidence of twisting features in the fibrils, both in 2D class averages or in the raw micrographs. A selection of 338,457 fibrils showing signs of internal features were re-extracted unbinned (with a 285 Å box size) and subsequent 2D classification showed clear layer lines in the class averages with a regular ~4.8 Å spacing, consistent with a cross-β structure in the fibrils (SI Appendix Figure S5C). To generate the intensity plots in SI Appendix Figure S5D, the grey values were measured along a straight line drawn parallel to the fibril axis for a single class average image in Fiji/ImageJ (11), with two examples shown for different class averages. 3D classification using a featureless cylinder as a starting template and helical twist searches around 359.5-360.0˚ did not yield any promising models to pursue further.

Rate-zonal density-gradient ultracentrifugation

The protocol was adapted from (12). For each experiment sucrose was dissolved to 50, 40, 30, 20 and 10 % (*w/v*) into the appropriate buffer at the pH used for the ThT assays. 750 µL of each solution was then layered into an OptiSeal polypropylene centrifuge tube (Beckman Coulter 361621) by pipetting equal amounts of each solution gently down the side of the tube with the 50% (*w/v*) solution first, to create a discrete sucrose gradient. 750 µL of protein-containing sample was added last. The sample was then subjected to ultracentrifugation at 113,000 x *g* for 4 h in a TLA110 rotor. 150 µL volumes were taken from the top down. Only the middle 150 µL from each sucrose fraction was analysed. Fractions were imaged by SDS PAGE and negative stain TEM as described above, except that the grids were held in each water droplet for 30 sec. The rate at which a particle moves through a solvent during centrifugation, the settling velocity, is defined by Stokes' law (equation 1):

Equation 1.

$$V = \frac{(p - p_0) \times g \times D^2}{18N_0}$$

where p is the particle density, p_0 is the solvent density, g is the acceleration force, D is the particle size and N_0 is the solvent viscosity.

When the particle density is larger than the solvent density, as is the case for the differential pelleting assay, the settling velocity is proportional to the particle size. However, in a rate-zonal density-gradient experiment, solvents with a variety of densities are used. When the solvent density becomes equal to the particle density ($p = p_0$), V = 0 and the particle will stop moving through the solvent. Equilibration over a long period of time (4 h here) will result in the separation of particles by density.

Liposome formation

25 mg of dry DMPS powder (Avanti polar lipids) was resuspended in 1 ml of 80:20 v/v chloroform and methanol. A film of lipid was created in a round bottom test tube by evaporating off the solvents using a stream of nitrogen followed by overnight vacuum desiccation. Where possible the following steps were performed above the lipid transition temperature (T_m) of DMPS. The lipid film was resuspended in 890 µl of 20 mM sodium phosphate buffer, pH 6.5, briefly vortexed for 30 s and incubated for 30 minutes. The multilamellar vesicles were then subject to five freeze thaw cycles before being extruded by being passed through a 100 nm membrane 21 times. The resulting liposomes were stored at 4℃ and were used within 48 h.

Fibril formation in the presence of liposomes

Amyloid formation in the presence of DPMS LUVs was monitored using ThT fluorescence under quiescent conditions, using 50 μM initial monomer, 30℃, with no beads in 20mM sodium phosphate buffer, pH 6.5. All other parameters were the same as in the fibril formation assays as described in the absence of liposomes.

Liposome binding by circular dichroism

Far-UV CD spectra were acquired in 1-mm path length quartz cuvettes (Hellma) using a ChirascanTM plus CD Spectrometer (Applied Photophysics). CD spectra were acquired using a 1-nm bandwidth and 1-s time step, and data were collected at 1-nm increments at 30 °C. Three scans (190−260 nm) were acquired and averaged per sample per repeat of the experiment. Binding of γSyn variants to liposomes was calculated using a previously described (13) fitting protocol and equation 2.

Equation 2

$$X_B = \left(\frac{\left([Syn] + \frac{x[Syn]}{L} + K_D\right) - \sqrt{\left([Syn] + \frac{x[Syn]}{L} + K_D\right)^2 - \frac{4x[Syn][Syn]}{L}}}{2[Syn]}\right)$$

Where x is the concentration of DMPS divided by the concentration of synuclein protein, X_B is the fraction of bound protein calculated by subtracting the CD signal of free protein from the observed CD signal and dividing that by the difference in CD signal of protein in the free and saturated bound state and L is the number of lipids interacting with one protein monomer.

Cell culture experiments

Plasmid constructs for expression of four γSyn variants in eukaryotic cells were generated by PCR from corresponding plasmids in pCS19 vector (see above) using the primers: forward 5′-CCCCTCGAGACAACCAAAATGGATGTCTTCAAGA-3′ and reverse 5′-CCGAATTCTCTAGATCAGTCTCCC-3′. The PCR fragments were digested with XhoI/EcoRI, cloned into pcDNA3.1 mammalian expression vector and inserts were verified by DNA sequencing.

Human neuroglioma cells (H4) were maintained in Opti-MEM I Reduced Serum Medium (Life Technologies- Gibco, Carlsbad, CA, USA) supplemented with 10% (*v/v*) Fetal Bovine Serum Gold (FBS) (PAA, Cölbe, Germany) and 1% (*w/v*) penicillin-streptomycin (PAN, Aidenbach, Germany). The cells were grown at 37°C in an atmosphere of 5% CO_2.

Twenty-four hours prior to transfection, cells were plated in different well-plate formats, at a density of 50–60% confluency prior to transfection. The day after, cells were transfected with

equimolar ratios of plasmids with cDNA encoding the different variants of human γSyn. Transfections were performed with calcium phosphate. Briefly, 3 h prior to transfection, fresh cell medium was added to the cells. DNA was diluted in 1 × HBS buffer (25 mM 4-(2-hydroxyethyl)-1-piperazineethanesulfonic acid, 140 mM NaCl, 5 mM KCl, 0.75 mM Na_2HPO_4 $2H_2O$, 6 mM dextrose, pH 7.1) and 2.5 M $CaCl_2$ solution was added dropwise with vigorous mixing. After incubation for 20 min, the mixture was added dropwise to the cells. After 16 h the cells were fed with fresh medium.

For immunocytochemical analyses (ICC) cells were plated on 13 mm glass coverslips in 24-well plates. Forty-eight hours after transfection, H4 cells were washed with PBS and fixed with 4% (*v/v*) paraformaldehyde (PFA) for 20 min at room temperature (RT), followed by a permeabilisation step with 0.5% (*v/v*) Triton X-100 (Sigma-Aldrich, St. Louis, MO, USA) for 20 min at RT. After blocking in 1.5 % (*v/v*) goat serum (PAA, Cölbe, Germany)/DPBS for 1 h, cells were incubated overnight at 4 °C with primary rabbit anti-human γSyn antibody (14) diluted 1:500, following washes and incubation with secondary antibody (Alexa Fluor 488-donkey anti-rabbit IgG diluted 1:5000 (Life Technologies- Invitrogen, Carlsbad, CA, USA)) for 2 h at RT. Nuclei were counterstained with 4′6′-diamidino-2-phenylindol (DAPI) (Sigma-Aldrich, D8417) (1:5000 in DPBS) for 10 min. After a final wash, coverslips were mounted by using Mowiol (Sigma Aldrich, St. Louis, MO, USA) and subjected to fluorescence microscopy. Images were acquired using a 63× objective and analysed using LAS AF v.2.2.1 (Leica Microsystems) software. The percent of cells containing γSyn aggregates within the population of successfully transfected cells was then determined by counting. At least 100 transfected cells were assessed per variant in each of 4 independent experiments. The percent of cells containing large (>2 µm) vs, small (<2 µm) γSyn aggregates were also assessed. Statistical analyses of the data were performed using the one-way ANOVA with Turkey post-hog test for multiple comparisons or Student's t-test for independent variables. Differences were considered statistically significant at $^{*}p < 0.05$.

Statistical analyses of the data were performed using the one-way ANOVA or Student's t-test for independent variables. The data are presented as mean ± standard deviation and represent the results from at least four independent experiments. Differences were considered statistically significant at $^{*}p < 0.05$.

Fly stocks

Flies were maintained on cornmeal-yeast-molasses medium. Flies were reared and maintained at 25°C in a 12 h light:dark cycle. The following fly stocks were used: $elav^{C155}$-Gal4 (BSDC:458); GMR-Gal4 (BDSC:1104); $VGlut^{OK\text{-}371}$-Gal4 (BDSC:26160); UAS-mCD8::GFP (15). Transgenic flies expressing *SNCG* variants' cDNA were inserted into a pJFRC5-5XUAS-

IVS-mCD8::GFP (a gift from Gerald Rubin, Adgene plasmid # 26218) expression vector using standard PCR-based cloning. Embryo injections targeting the pJFRC5 vector independently to AttP40 and AttP2 landing sites was conducted via a commercial service (BestGene, USA).

For survival experiments, transgenes for UAS-SNCG variants or control UAS-mCD8::GFP were expressed under transcriptional regulation of Elav-Gal4. Vials containing approximately 10 flies were passaged to fresh food and survival censused on every second day.

Eye phenotype

UAS-SNCG variants or control UAS-mCD8::GFP transgenes were crossed to eye specific GMR-Gal4. Flies were aged up to 3 weeks at 25°C (n=10), at which point each were collected and frozen at -20°C in 1.5 mL microcentrifuge tubes, for imaging. Eyes were examined and images captured using Zeiss Stemi 508 with Zeiss AxioCam ERc 5s Rev.2.

Rapid iterative negative geotaxis (RING)

We conducted a modified version of RING assay (16). Flies expressing UAS-SNCG variants or control UAS-mCD8::GFP were reared and maintained at 25°C. For RING analysis groups of 10 flies per genotype were passaged into the empty vials and left to acclimatize for 10 min. Vials were mounted into a custom-built frame composed of a vial carrier and vertical rails, allowing parallel analysis of six vials from a consistent height (20 cm) and impact. Digital images were collected for analysis at 10 sec post-impact (Panasonic Lumix DC-F782). The assay was repeated five times. Images were analysed in ImageJ, with height climbed of individual flies recorded. The average distance travelled by all flies in a single vial, averaged across five consecutive RING trials is considered 1 technical replicate.

Immunofluorescent staining

Drosophila heads were removed and fixed for 20 min in 4% (*v/v*) paraformaldehyde (PFA) in phosphate buffered saline containing 0.1% (*v/v*) Triton-x100 (PBS-Tx). Whole brains were dissected and fixed for a further 20 min in 4% (*v/v*) PFA in PBS-Tx. Brains were blocked in 10% (*v/v*) goat serum in PBS-Tx for 30 min, then incubated with primary rabbit polyclonal anti-human γSyn-specific SK109 antibody (17) overnight at 4°C. Following washing with PBS-Tx, brains were incubated overnight with fluorescent secondary antibody (1:200, goat anti-rabbit IgG Alexa Fluor 488, ThermoFisher A-11008). Maximal orthogonal z-projection fluorescent micrographs were collected using a Zeiss Cell Observer Spinning Disc confocal microscope using Zen Blue software (Version 2.6).

Supplementary results

Expression of γSyn variants in neurons has a subtle effect on the phenotype of transgenic *Drosophila* flies

To elucidate if expression of the γSyn M38/E110, M38/V110, I38/E110 or I38/V110 variants results in aggregate formation or causes an effect in an organismal context, we produced transgenic *Drosophila* lines with the human *SNCG* gene variants under control of 5xUAS elements and crossed them with the driver lines expressing Gal4 in the developing eye cells (GMR-Gal4), pan-neuronal (Elav-Gal4) or in glutamatergic neurons (OK371-Gal4). Similar levels of expression of human γSyn variants were detected in four transgenic flies by Western blotting (SI Appendix Figure S9A). Ectopic expression of neurodegenerative disease associated proteins in photoreceptor neurons during development and ageing can result in morphological disruption of the *Drosophila* compound eye. Eye morphology of all four γSyn lines was comparable to non-transgenic control flies, with no abnormal developmental changes nor degenerative phenotypes emerging up to 3-weeks post-eclosion (SI Appendix Figure S9B). To determine the aggregation properties of variant transgenes, we also immunostained for γSyn aggregates in glutamatergic neurons in the optic lobe of 5-week post-eclosion adult flies (SI Appendix Figure S9C). Distribution of γSyn was comparable in all transgenic lines, present diffusely within cytosol and as small puncta throughout glutamatergic neuron soma and neurites. For these assays it should be considered that a toxic effect might be predominantly driven by the formation of oligomers rather than fibrils. In fact, the formation of amyloid fibrils could also be a protective mechanism to eliminate toxic oligomers as it has been reported previously for αSyn (18, 19). The locomotor activity of flies with pan-neuronal expression of γSyn variants was tested in the negative geotaxis (RING) assay at the age of 20 and 50 days post-eclosion (dpe). When age-related changes were compared to that of a control of membrane-tethered GFP-expressing flies (performance on 50 dpe 93.7 ± 9.94% from the performance at 20 dpe), a significant decrease of locomotor activity was observed for lines expressing γSyn I38/V110 (performance 50/20 dpe 49.3 ± 2.56%), M38/V110 (performance 50/20 dpe 52.3 ± 1.21%) and particularly for the line expressing M38/E110 (29.7 ± 4.15%), while the decrease for the line expressing I38/E110 (performance 50/20 dpe 64.1 ± 7.47%) did not reach statistical significance (SI Appendix Figure S9D). Although reduced mobility of ageing flies may be a sign of developing pathology, neither of the four γSyn transgenic lines displayed compromised longevity in survival assays (SI Appendix Figure S9E). Kaplan-Meier analysis revealed that with exception of flies expressing γSyn I38/E110 line, whose survival was not significantly different from that of control flies expressing GFP,

flies of other three lines survived marginally, but statistically significantly better than control flies ($p<0.001$ for both Mantel-Cox log-rank and Gehan-Breslow-Wilcoxon tests).

Supplementary figures

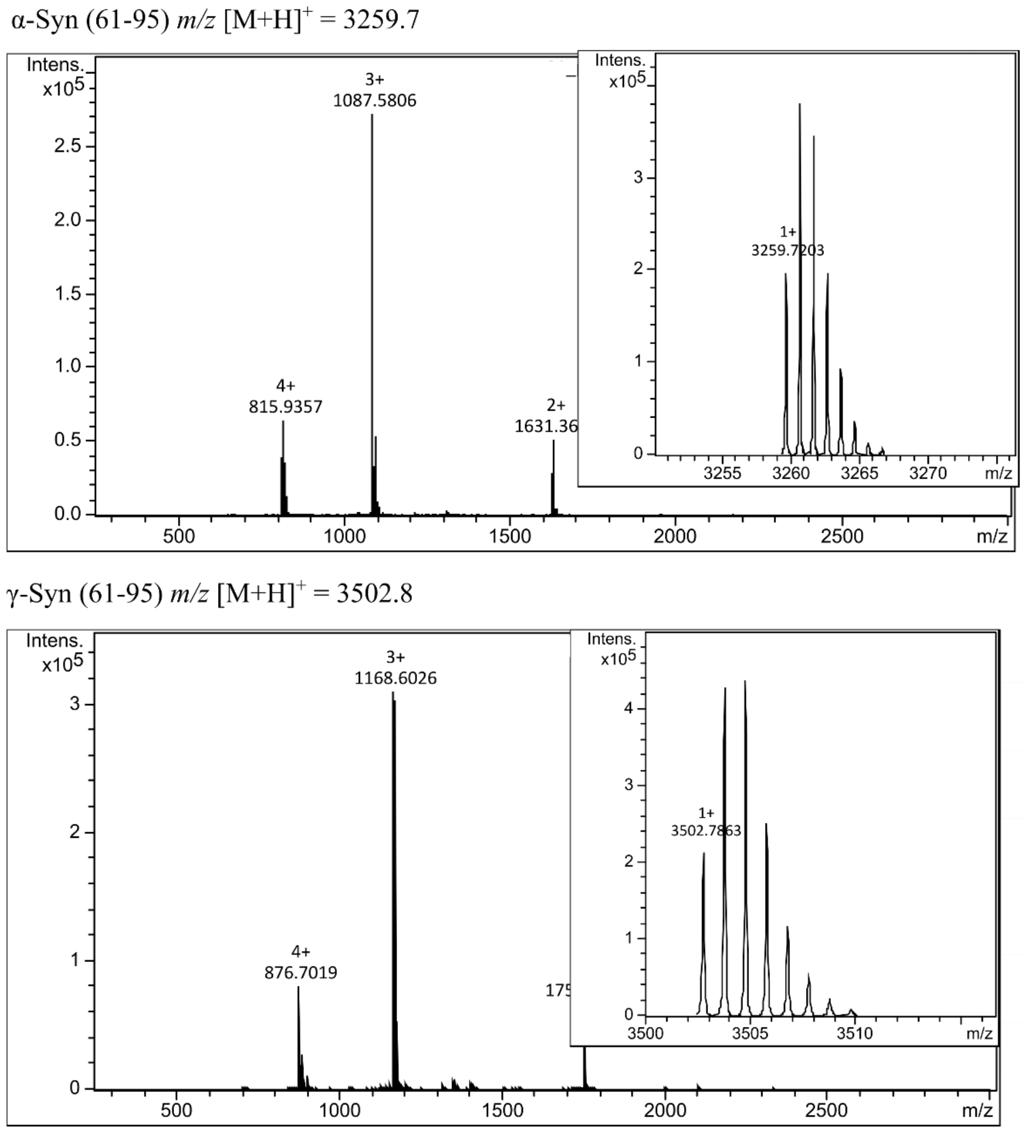

Supplementary Figure S1. Mass spectra of synthesised NAC peptides. Intact mass spectra of the αSyn and γSyn NAC peptides. Insets show the deconvoluted mass spectra demonstrating m/z peaks corresponding to the charge states of the peptides and that the monoisotopic mass matches that of the expected peptides (αSyn NAC expected mass m/z [M+H]+ = 3259.717 Da observed mass [M+H]+ = 3259.720 Da, γSyn expected mass m/z [M+H]+ = 3502.776 Da observed mass [M+H]+ = 3502.786 Da).

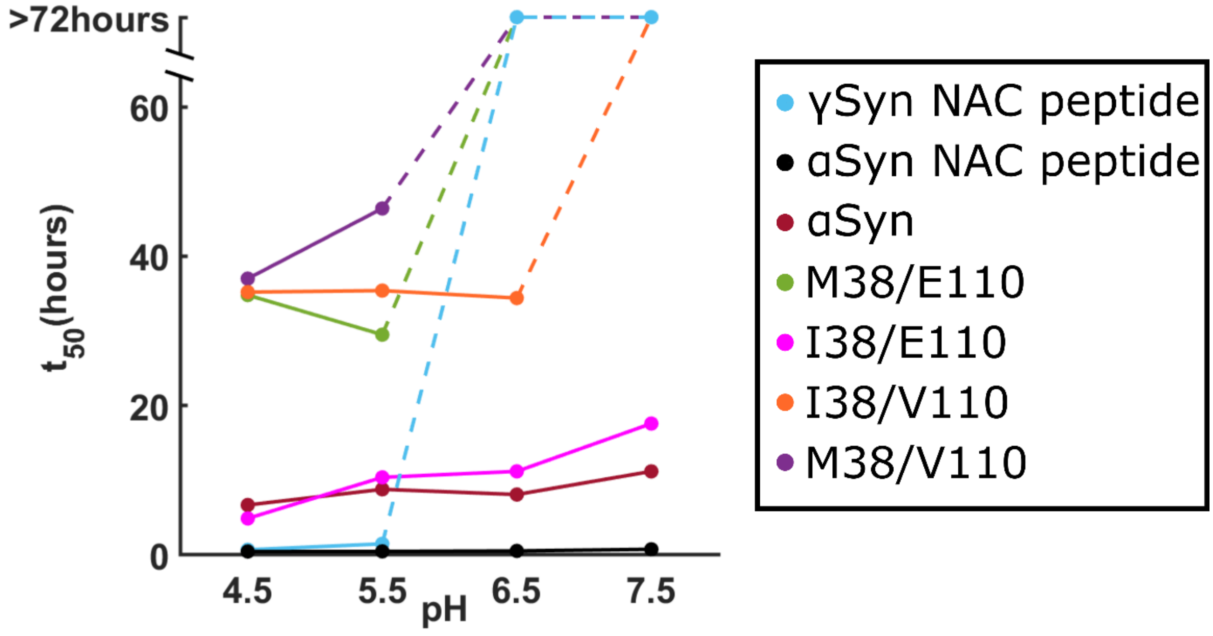

Supplementary Figure S2. The pH dependence of αSyn and γSyn fibril assembly compared with the αSyn and γSyn NAC peptides. T_{50} of amyloid fibril formation vs pH for the γSyn NAC peptide (blue), the αSyn NAC peptide (black), αSyn (maroon), γSyn M38/E110 (green), γSyn I38/E110 (pink), γSyn I38/V110 (orange) and γSyn M38/V110 (purple). Fibril formation of the γSyn NAC peptide is highly pH dependent and an amino acid substitution of a Met to an Ile at residue 38 of γSyn results in a shift in the pH dependence. Fibril assembly was monitored for 72 h, and so dotted lines are used here to represent samples where an enhanced ThT signal was not observed within 72 h.

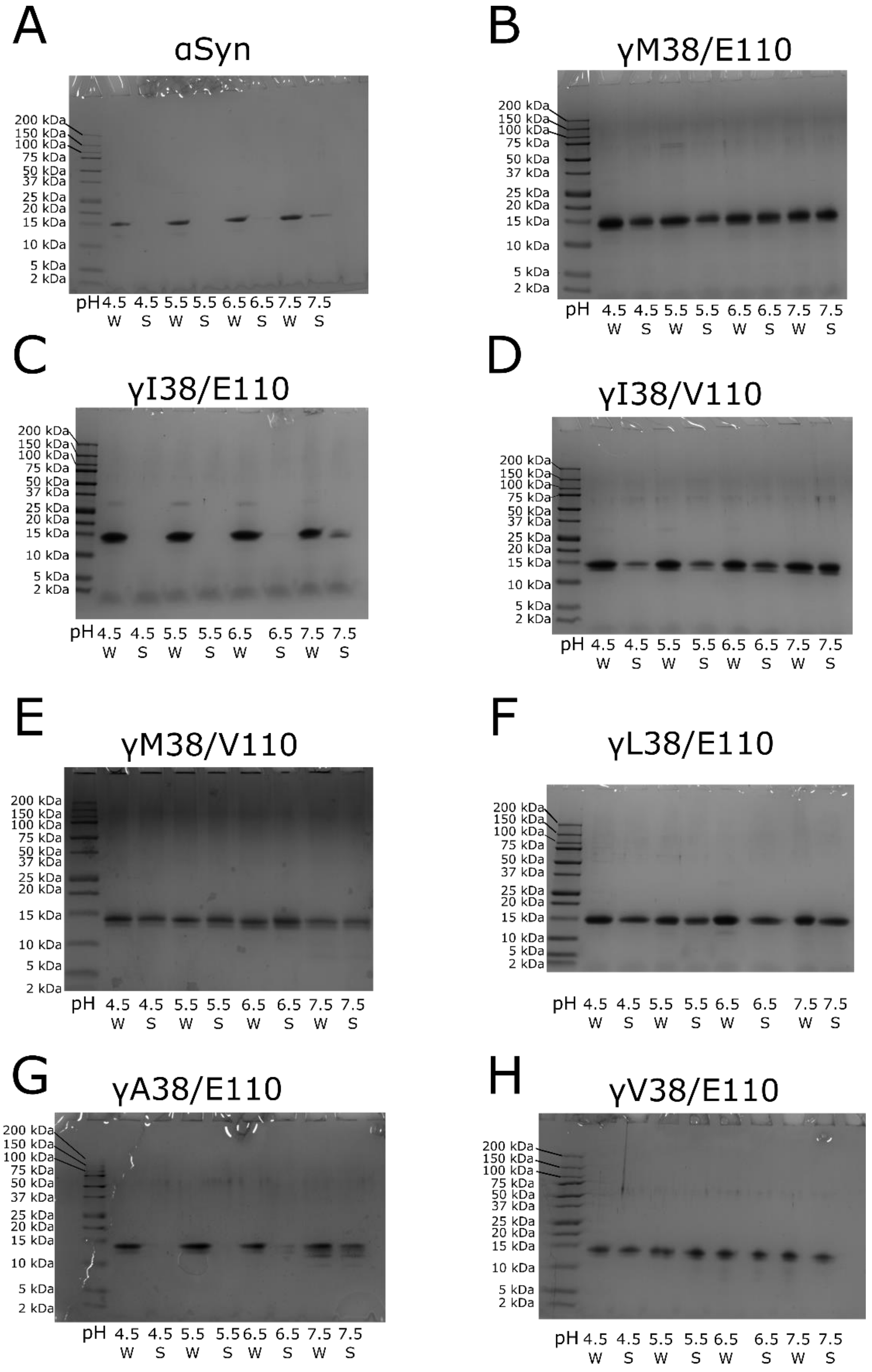

Supplementary Figure S3. Pelleting assay results. Representative SDS PAGE gels of the end points of ThT assays demonstrating the whole (W) and soluble (S) fractions after ultracentrifugation at 100,000 x *g* for 30 min. **(A)** αSyn, (B) γSyn M38/E110, **(C)** γSyn I38/E110, **(D)** γSyn I38/V110, **(E)** γSyn M38/V110, **(F)** γSyn L38/E110, **(G)** γSyn A38/E110, **(H)** γSyn V38/E110.

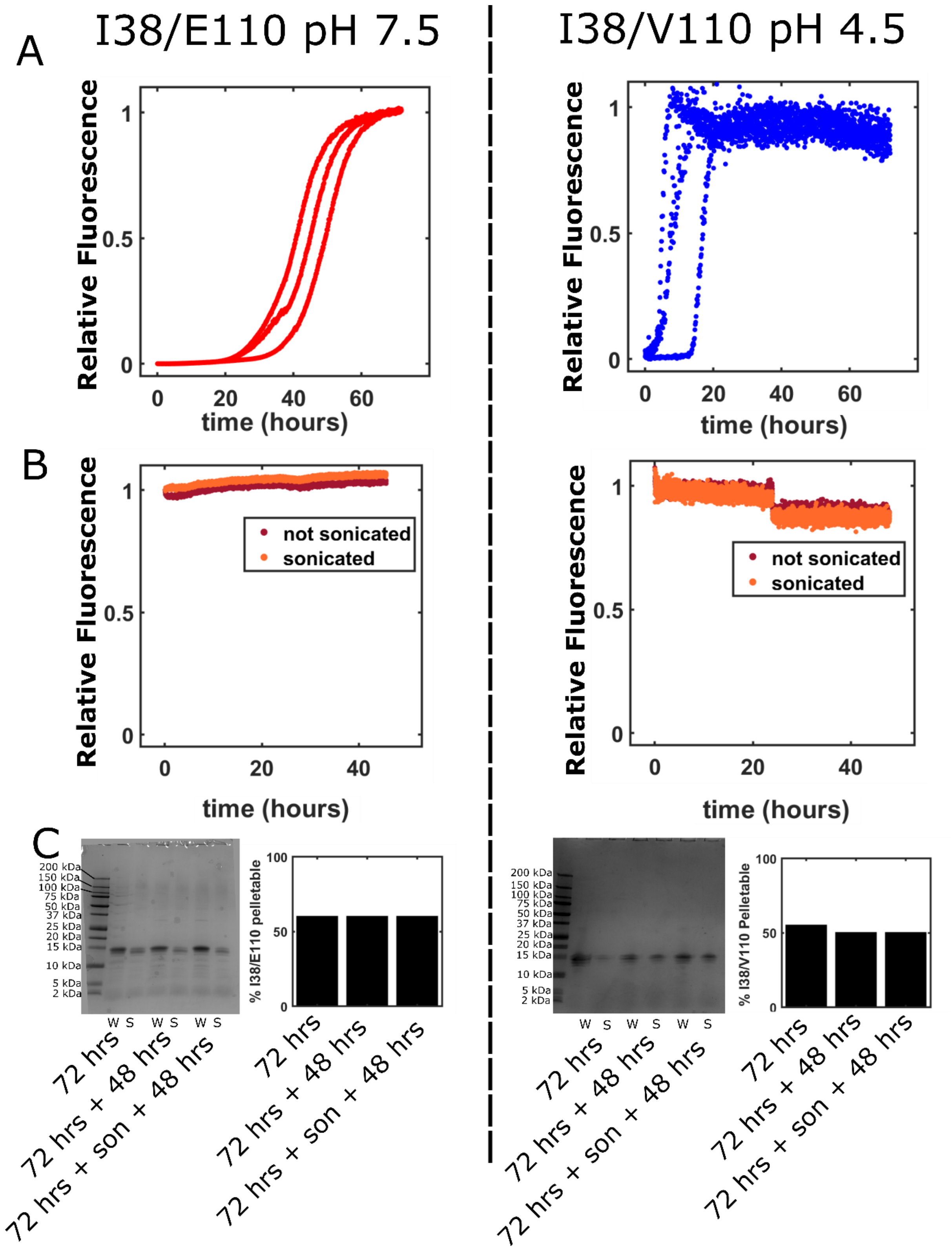

Supplementary Figure S4. γSyn variant fibril formation has reached an equilibrium after 72 h. (A) Fibril formation, monitored by ThT fluorescence of I38/E110 at pH 7.5 (left, red) and I38/V110 at pH 4.5 (right, blue) (with beads and shaking) for 72 h. Three replicates are shown. Note that the plate reader was restarted after 24 h, which resulted in a small change in

fluorescence signal. **(B)** Kinetics of fibril formation of I38/E110 at pH 7.5 and I38/V110 at pH 4.5 monitored by ThT fluorescence in which samples taken after 72 h of incubation with beads and shaking (A) were then either subjected to sonication (orange) or were not sonicated (brown) and then incubated for a second 48 h period under quiescent conditions. Three replicates of each are shown (some of which are overlapping). **(C)** Pelleting assay demonstrating that after sonication and an additional 48 h incubation no further pelletable material is formed, regardless of whether the sample had been sonicated.

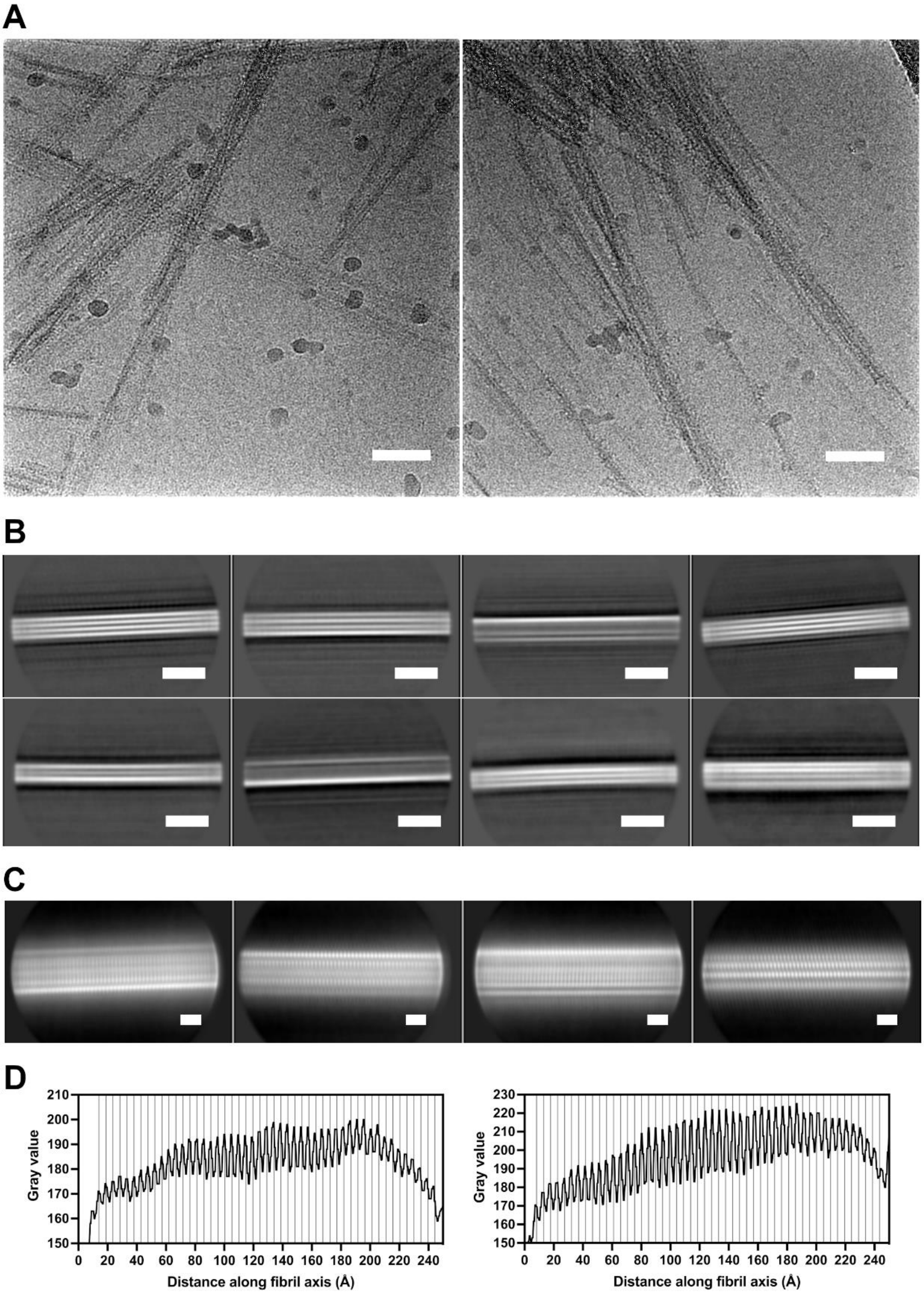

Supplementary Figure S5. Cryo-TEM of γSyn I38/E110 fibrils. (A) Two example regions of cryo-electron micrographs of fibrils of γSyn I38/E110 formed at pH 6.5 and 20 mM ionic strength with continuous shaking at 600 rpm in a 1.5 mL microfuge tube for 14 days (scale bars represent 50 nm). **(B)** 2D class averages from 2x binned segments show untwisted fibril

morphologies (scale bars represent 10 nm). **(C)** Unbinned 2D class averages, in which the ladder typical for cross-β structures can be clearly seen (scale bar represents 20 Å). **(D)** Plot of grey value along the length of two representative unbinned 2D class averages (shown in (C)) demonstrating that the peaks are aligned with the 4.8 Å repeating units (vertical lines) classical of cross-β amyloid.

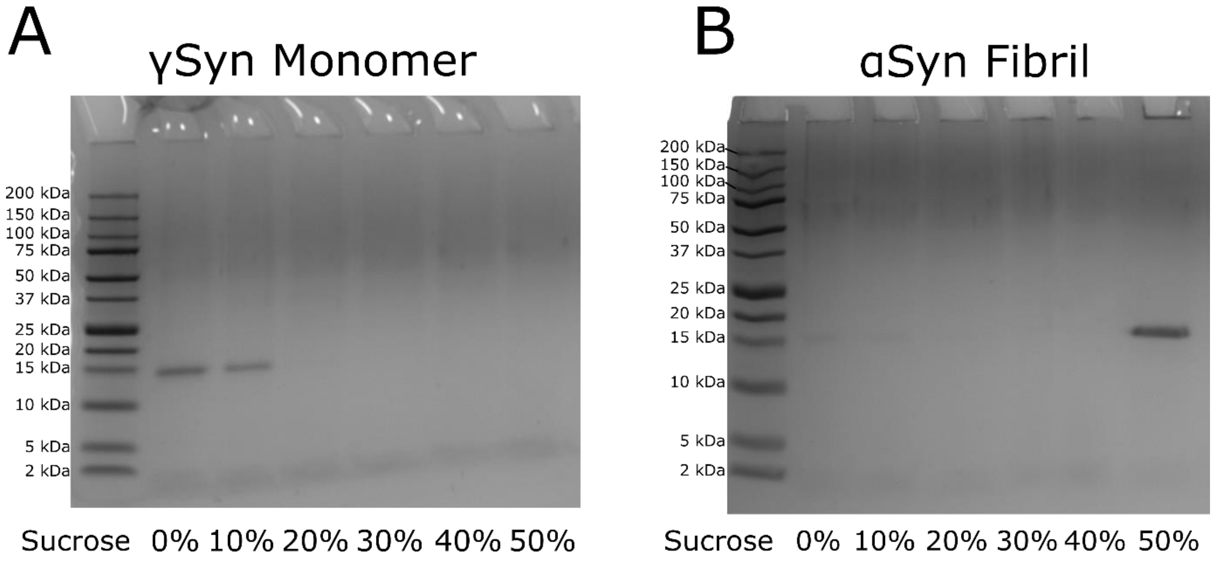

Supplementary Figure S6. Rate-zonal density-gradient ultracentrifugation of γSyn (M38/E110) monomers and αSyn fibrils on a discontinuous sucrose gradient. Representative SDS PAGE gels of **(A)** freshly dissolved γSyn M38/E110 monomers or **(B)** preformed αSyn fibrils, both at pH 7.5. The results show fractions taken after ultracentrifugation at 113,000*g* for 4 h in the presence of a discrete sucrose gradient.

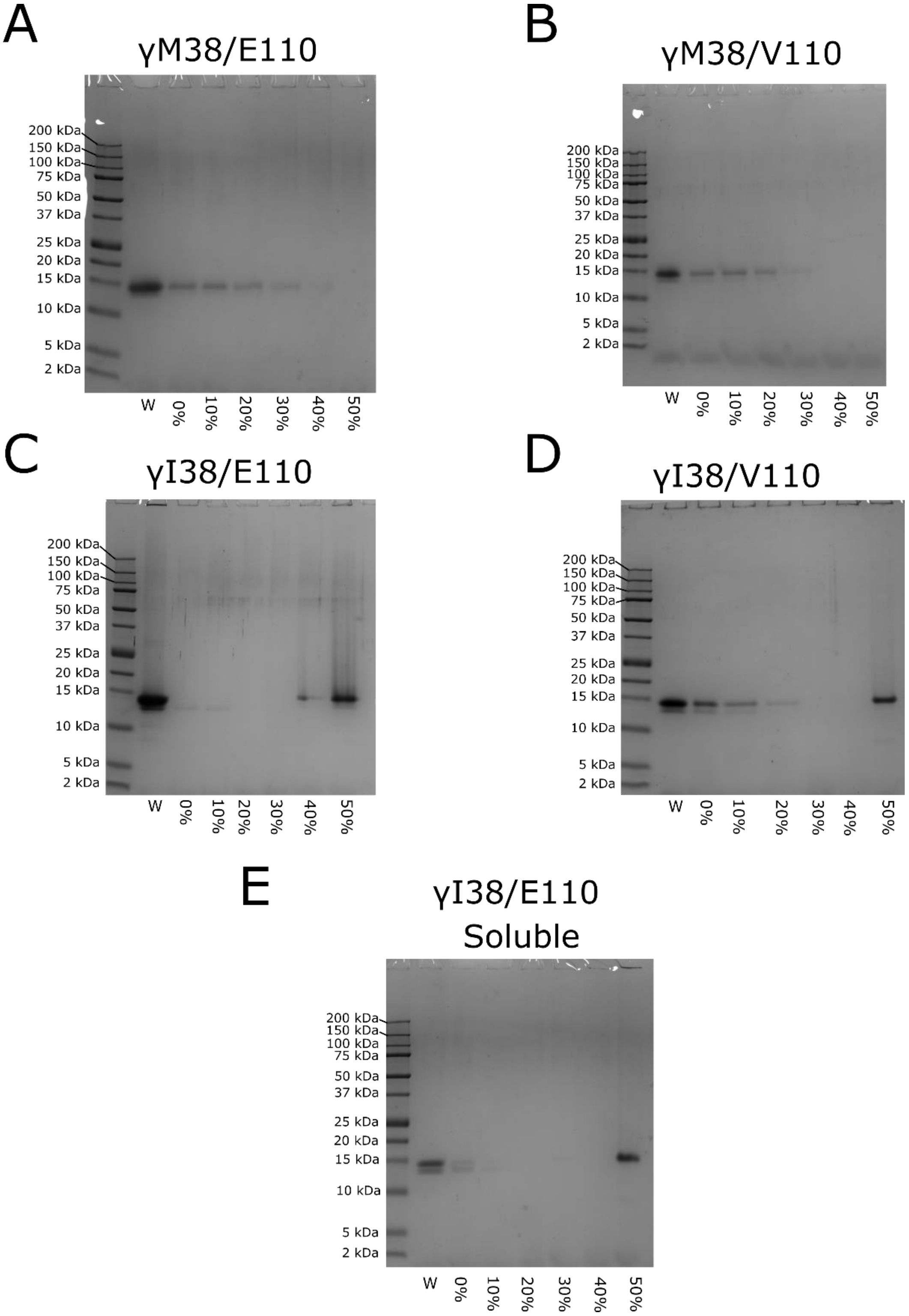

Supplementary Figure S7. Separation of monomers, oligomer and fibrils using rate-zonal density-gradient centrifugation with a discontinuous sucrose gradient. Representative SDS PAGE gels of the end points of ThT assays at pH 7.5 demonstrating fractions after ultracentrifugation at 113,000*g* for 4 h in the presence of a discrete sucrose

gradient (**A-D**), or after ultracentrifugation at 100,000*g* for 30 min to remove fibrils prior to addition to the sucrose gradient (**E**). The gels were loaded in the following order: ladder, whole sample (W), and the 0%, 10%, 20%, 30%, 40%, 50% (*w/v*) sucrose fractions. **(A)** γSyn M38/E110, **(B)** γSyn M38/V110, **(C)** γSyn I38/E110, **(D)** γSyn I38/V110, **(E)** γSyn I38/E110 supernatant after a pre-spin at 100,000*g* to remove fibrils.

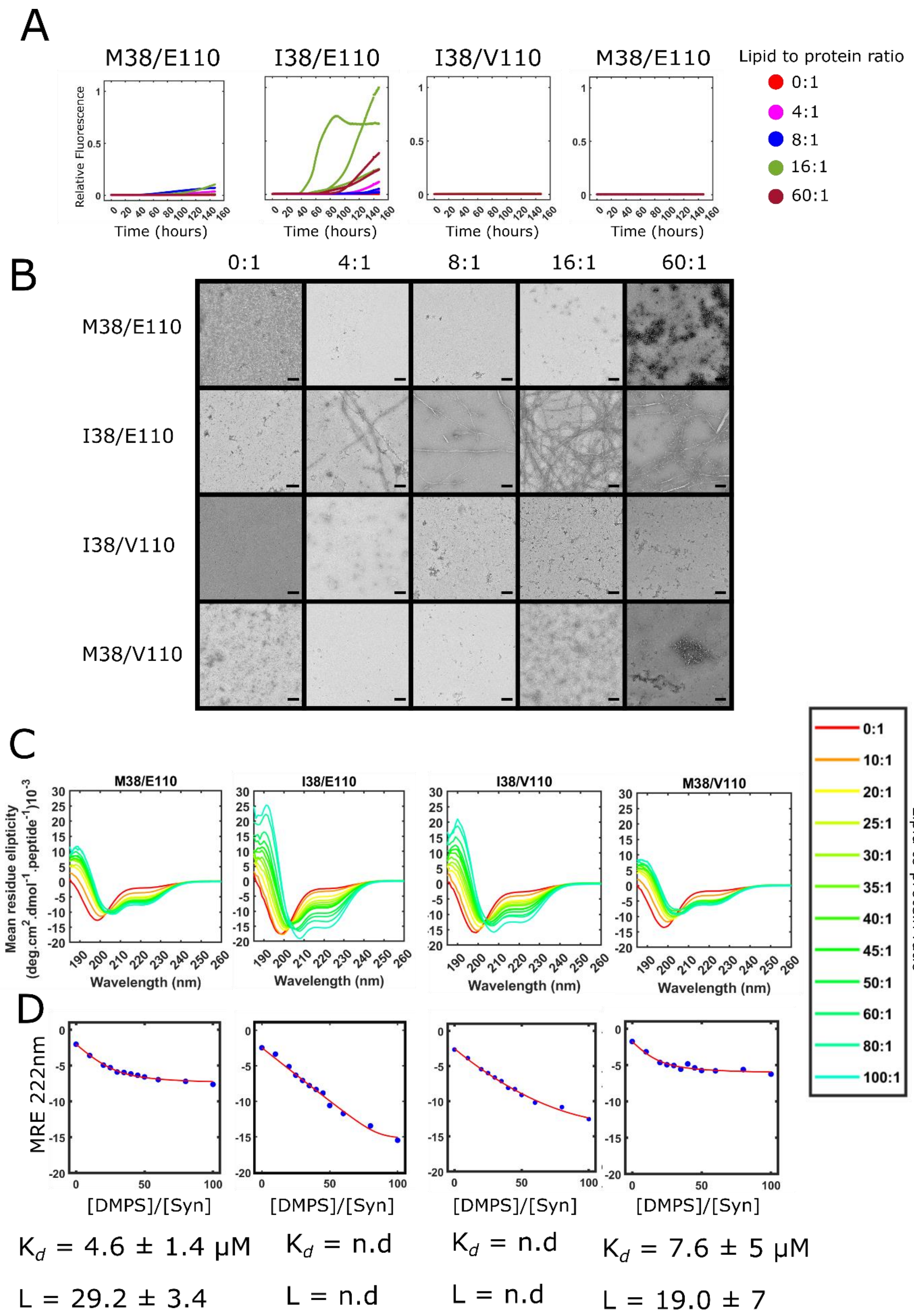

Supplementary Figure S8. I38 enables amyloid fibril assembly in the presence of liposomes, despite weaker binding to the lipid surface. (A) ThT fluorescence monitoring the incubation of γSyn variants at 0:1 (red), 4:1 (pink), 8:1 (blue), 16:1 (green) and 60:1

(maroon) LPRs with 100 nm DMPS liposomes over 150 h. **(B)** Negative stain TEM images of γSyn variants after 150 h incubation with 100 nm DMPS liposomes at 0,4,8,16 and 60:1 LPR, as indicated. **(C)** Far UV CD spectra of 25 μM γSyn variants mixed with 100 nm DMPS liposomes at 0, 10, 20, 25, 30, 35, 40, 45, 50, 60, 80 and 100:1 LPR. **(D)** The MRE at a wavelength of 222 nm for each LPR. The binding curve (in red) was fitted to find the K_d and L values (not possible for the I38 proteins which did not saturate at any of the lipid to protein ratios).

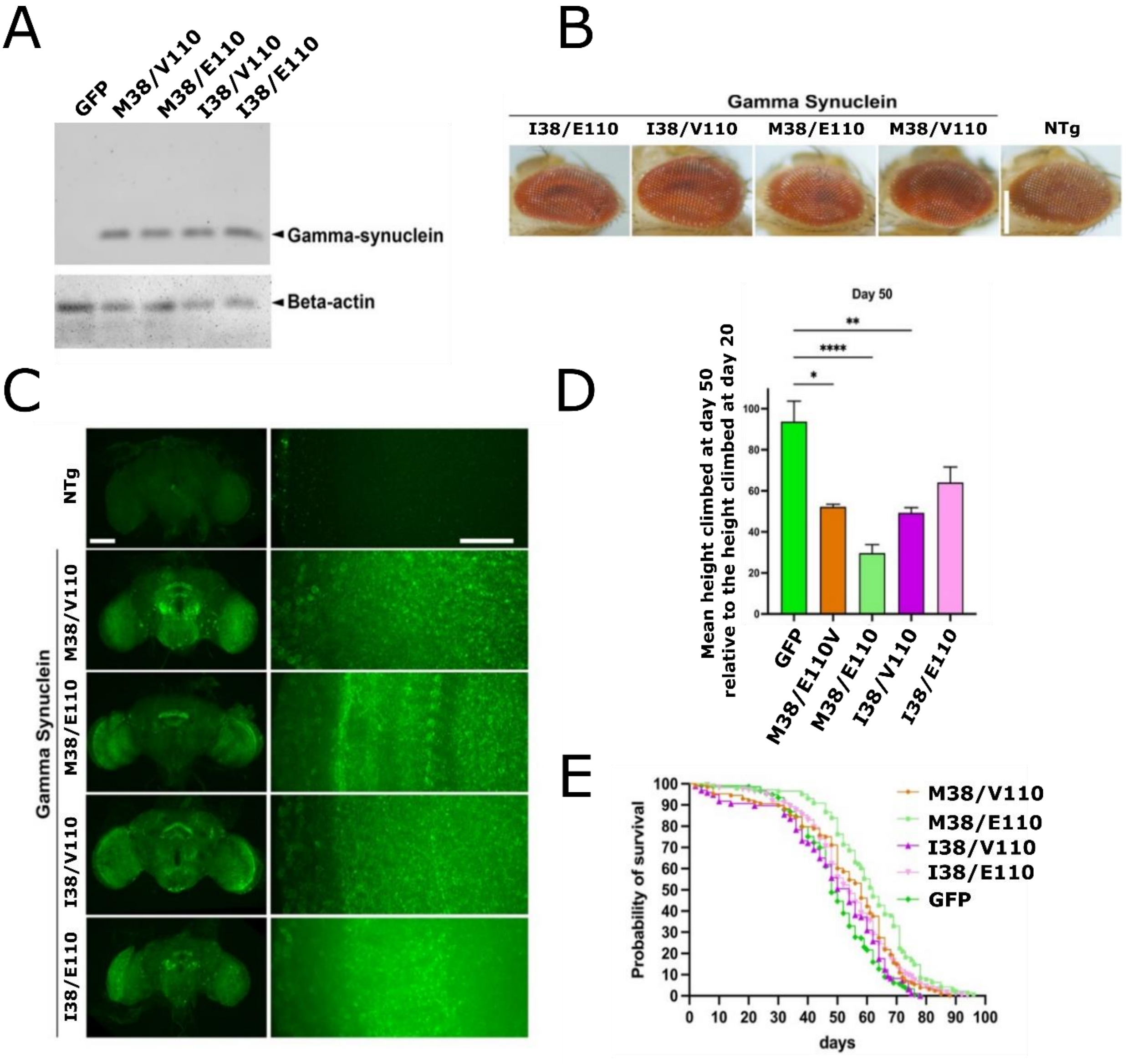

Supplementary Figure S9. Analysis of *Drosophila melanogaster* expressing human γSyn variants in neurons. (A) Western blot demonstrates equal levels of γSyn in total head lysates of adult flies of lines expressing the four γSyn variants under transcriptional control of pan-neuron promoter Elav-Gal4. **(B)** Expression of γSyn variants in the eye (GMR-Gal4) did not cause degenerative phenotypes up to 3 weeks post-eclosion (scale bar = 200μm). **(C)** Immunofluorescent labelling for human γSyn variants expressed in glutamatergic neurons (OK371-Gal4) revealed comparable diffuse and punctate staining throughout the brain, with no evidence of large protein inclusions. Low magnification (left) shows whole brain (scale bar = 100 μm), high magnification (right) shows optic lobe (scale bar = 20 μm). **(D)** A significant decline in RING behavioural assay performance was observed in transgenic flies expressing all four γSyn variants pan-neuronally (Elav-Gal4) between 20 and 50 dpe, compared with the GFP control. The bar chart shows the performance at day 50, expressed as percent of the

average height climbed normalised to the average height climbed by the same set of flies of each line at the age of 20 days as 100%. (*p<0.05; **p<0.01; ****p<0.0001, Kruskal-Wallis ANOVA with post-hoc Dunn's test, n=6 groups of flies per genotype). **(E)** Kaplan-Meier curves analysis revealed that with the exception of γSyn I38/V110, pan-neuronal expression of γSyn variants modestly improve survival of flies compared with GFP expressing controls (p<0.001 for both Mantel-Cox log-rank and Gehan-Breslow-Wilcoxon tests; n=128 for γSyn M38/E110V, n=142 for M38/E110, n=97 for I38/V110, n=277 for I38/E110, n=213 for GFP).

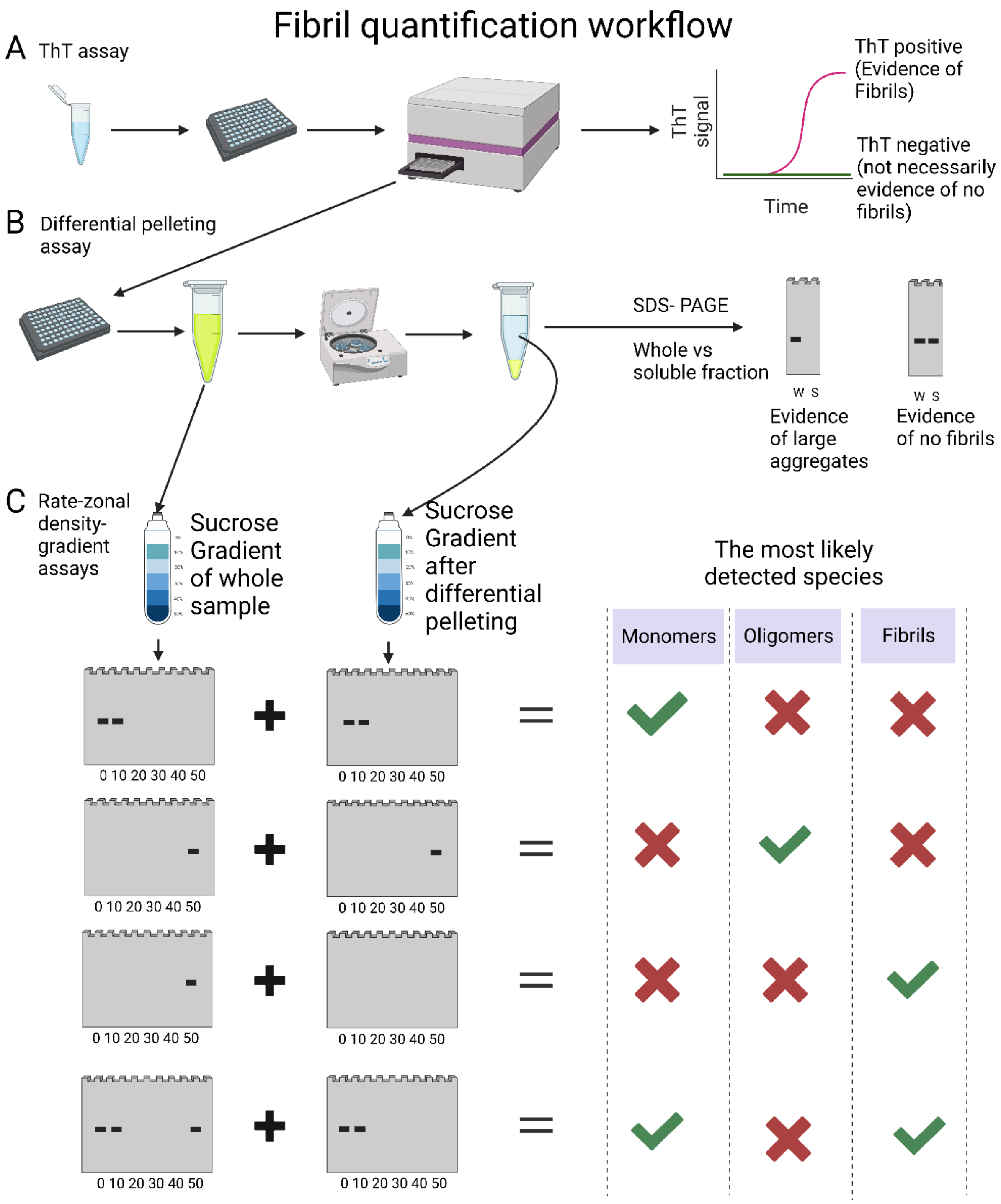

Supplementary Figure S10. A workflow for detecting and quantifying aggregating species. A schematic diagram demonstrating the workflow for detecting different species. **(A)** A ThT assay in which fibrils may be detected if the yield is high enough and the fibrils formed bind ThT strongly enough to result in a detectable signal. The rate of fibril assembly can be quantified directly from the experiment, but not the fibril yield. **(B)** A differential pelleting assay in which a sample at the endpoint of a ThT assay is subjected to centrifugation at 100,000*g* for 30 min and the soluble fraction quantified using SDS-PAGE. The amount of fibril/large

aggregate can be quantified from this experiment and the absence of fibrils can be determined if the amount of protein in the whole sample is equal to that in the soluble sample. **(C)** Rate-zonal density-gradient ultracentrifugation. Since γSyn monomers equilibrate in the 0 and 10% (*w/v*) sucrose fractions (Supplementary Figure S7) we assume that all sample that equilibrates between 0 and 10% (*w/v*) sucrose is monomer. This assay can separate monomer from oligomer, but not dense oligomers from fibrils which both may pellet in the 50 % (*w/v*) sucrose fraction. Performing density gradient centrifugation in a discontinuous sucrose gradient subsequent to differential pelleting (which removes fibrils (20)) can thus be used to separate fibrils from other dense aggregates. Schematic diagrams of SDS PAGE gels are shown (all bands are considered to be of exactly equal intensity in these particular examples), alongside a table indicating how the example data would be interpreted. Created using biorender.com.

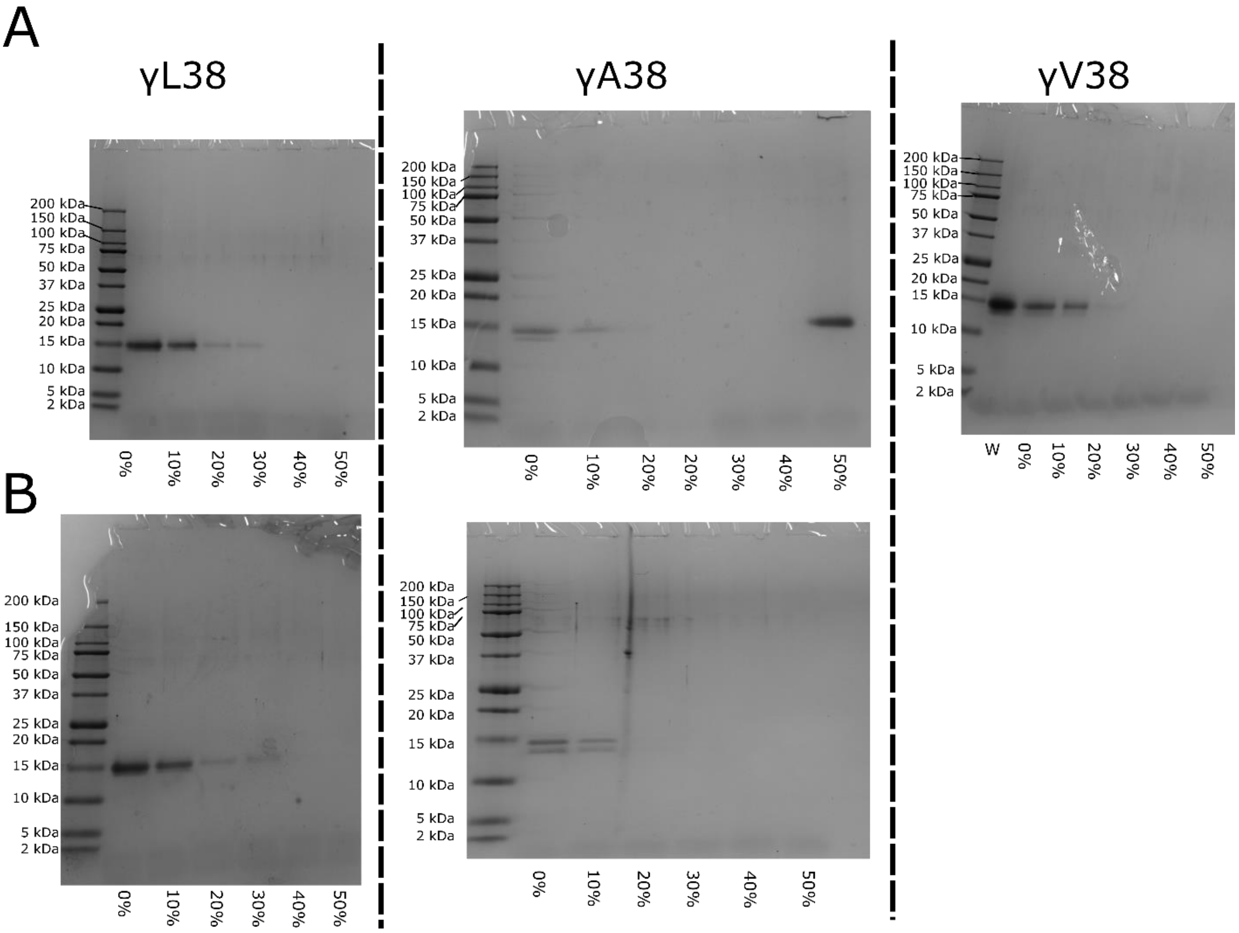

Supplementary Figure S11. Rate-zonal density-gradient ultracentrifugation using a discontinuous sucrose gradient of different γSyn variants. Representative SDS PAGE gels of γSyn L38, γSyn A38 and γSyn V38 **(A)** at the end points of ThT assays at pH 7.5 and **(B)** the soluble fraction subsequent to differential pelleting by ultracentrifugation at 100,000*g* for 30 min. The gels were loaded in the following order: ladder, 0%, 10%, 20%, 30%, 40%, 50% (*w/v*) sucrose fraction. All variants contained E110.

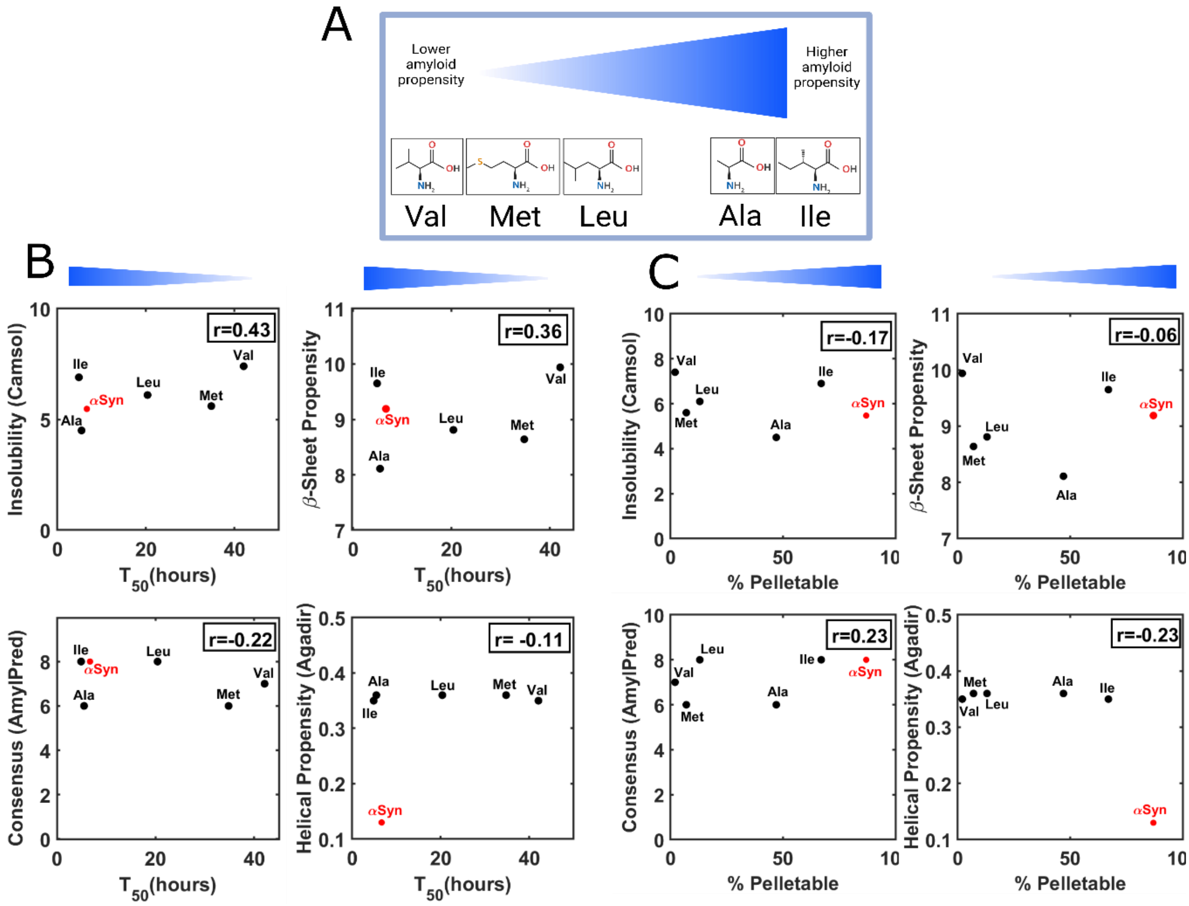

Supplementary Figure S12. The T_{50} of ySyn amyloid formation does not correlate with the properties of the protein dependent on the identity of residue 38. (A) Rank order of amyloid formation (T_{50}) of Met, Leu, Ile, Val and Ala at residue 38 (all contain E110). **(B)** Correlation of the T_{50} of amyloid formation (pH 4.5) with the solubility (top left) (CamSol (3)), β-sheet propensity (top right) (21), amyloidogenicity (bottom left) (AmylPred (22)), or helical propensity of the 20 amino acids starting at P1 and ending after P2 (bottom right) (Agadir (23)). A peptide sequence of this length was chosen as the seven residues in P1 alone are too short to determine whether or not a helix will form locally. **(C)**, As **(B)**, but for the % pelletable material at pH 7.5 (differential centrifugation at 100,*000g).* Where possible, all predicted values were calculated at pH 7.5 and 20 mM ionic strength. The values for αSyn are shown for comparison in each plot in red and were not included in the correlation calculation (Pearson's correlation).

Supplementary Tables

Table S1 Half times, lag times and pelletable material of αSyn and γSyn variants from ThT assays

Protein	Buffer pH	T_{50} (hours)	T_{lag} (hours)	% pelletable material
αNAC	4.5	**0.47**±0.03	**N/D**	**82.5**±2.5
	5.5	**0.49**±0.01	**N/D**	**85.5**±0.5
	6.5	**0.55**±0.05	**N/D**	**88.5**±1.5
	7.5	**0.77**±0.08	**N/D**	**90**±5
γNAC	4.5	**0.7**±0.1	**N/D**	**85**±0.2
	5.5	**1.5**±0.1	**N/D**	**87.5**±2.5
	6.5	**N/D**	**N/D**	**28**±13
	7.5	**N/A**	**N/A**	**0**
αSyn	4.5	**6.7**±0.3	**5.7**±0.3	**100**±0
	5.5	**8.8**±1.9	**7.7**±0.8	**98**±2
	6.5	**8.1**±0.4	**6.7**±0.4	**90**±3
	7.5	**11.2**±0.7	**7.4**±0.7	**87**±2
γM38/E110	4.5	**34.8**±4.7	**22.5**±3.6	**25**±9
	5.5	**29.5**±3.0	**12.6**±0.8	**27**±9
	6.5	**N/D**	**N/D**	**7**±2
	7.5	**N/A**	**N/A**	**7**±2
γI38/E110	4.5	**4.9**±0.8	**0.9**±0.5	**97**±2
	5.5	**10.4**±0.7	**3.3**±0.9	**95**±3
	6.5	**11.2**±1.2	**6.2**±0.6	**85**±13
	7.5	**17.6**±2.1	**13.7**±2.1	**67**±2
γI38/V110	4.5	**35.2**±4.6	**12.3**±3.6	**53**±4
	5.5	**35.4**±3.6	**13.2**±2.2	**38**±7
	6.5	**34.4**±5.6	**18.3**±2.8	**30**±10
	7.5	**N/D**	**N/D**	**10**±3
γM38/V110	4.5	**37.0**±1.4	**29.5**±1.1	**15**±3
	5.5	**46.4**±8.9	**39.2**±7.7	**8**±3
	6.5	**N/A**	**N/A**	**3**±2
	7.5	**N/A**	**N/A**	**5**±3
γL38	4.5	**20.4**±2.6	**15.7**±2.7	**23**±4
	5.5	**29.1**±4.4	**12.3**±0.3	**20**±3
	6.5	**36.4**±2.8	**25.7**±2.2	**13**±4
	7.5	**N/A**	**N/A**	**13**±3
γA38	4.5	**5.5**±0.4	**4.1**±0.3	**95**±0
	5.5	**9.3**±0.4	**5.6**±0.4	**92**±3
	6.5	**10.7**±0.4	**6.7**±0.4	**90**±3
	7.5	**34.5**±5.0	**27.4**±5.0	**47**±10
γV38	4.5	**42.1**±6.6	**33.0**±6.1	**15**±3
	5.5	**N/D**	**N/D**	**3**±2
	6.5	**N/A**	**N/A**	**3**±2
	7.5	**N/A**	**N/A**	**2**±2

T_{50} is the time taken to reach half of the maximum signal and T_{lag} is the x-intercept of the tangent to the inflection point of the sigmoidal curve. In cases where no ThT signal change was observed T_{50} and T_{lag} measurements are marked as not applicable (N/A). T_{50} and T_{lag} measurements were not determined (N/D) when assembly occurred too rapidly for a lag time to be determined, when the signal for the majority of replicates did not reach a plateau within 72 h preventing maximum signal determination, or where the signal change represented <10% of the sample determined by the pelleting assay. The standard errors about the mean of the T_{50} and T_{lag} values are presented. The percent material found in the pellet after ultracentrifugation at 100,000*g* (Methods) is shown (three biological replicates). Note that the L38, A38 and V38 γSyn variants all contained E110. All reactions were performed using 80 μM protein or peptide at 37℃ and shaking at 600 rpm in the presence of teflon beads.

Supplementary References

1. F. Sievers, *et al.*, Fast, scalable generation of high-quality protein multiple sequence alignments using Clustal Omega. *Mol. Syst. Biol.* **7**, 539 (2011).
2. G. G. Tartaglia, M. Vendruscolo, The Zyggregator method for predicting protein aggregation propensities. *Chem. Soc. Rev.* **37**, 1395–1401 (2008).
3. P. Sormanni, F. A. Aprile, M. Vendruscolo, The CamSol method of rational design of protein mutants with enhanced solubility. *J. Mol. Biol.* **427**, 478–490 (2015).
4. N. J. Anthis, G. M. Clore, Sequence-specific determination of protein and peptide concentrations by absorbance at 205 nm. *Protein Sci. Publ. Protein Soc.* **22**, 851–858 (2013).
5. C. P. A. Doherty, *et al.*, A short motif in the N-terminal region of alpha-synuclein is critical for both aggregation and function. *Nat. Struct. Mol. Biol.* **27**, 249–259 (2020).
6. C. Sengstag, The sequence of Saccharomyces cerevisiae cloning vector pCS19 allowing direct selection for DNA inserts. *Gene* **124**, 141–142 (1993).
7. N. Ninkina, *et al.*, Contrasting effects of α-Synuclein and γ-Synuclein on the phenotype of Cysteine String Protein α (CSPα) Null mutant mice suggest distinct function of these proteins in neuronal synapses. *J. Biol. Chem.* **287**, 44471–44477 (2012).
8. D. Kimanius, L. Dong, G. Sharov, T. Nakane, S. H. W. Scheres, New tools for automated cryo-EM single-particle analysis in RELION-4.0. *Biochem. J.* **478**, 4169–4185 (2021).
9. A. Rohou, N. Grigorieff, CTFFIND4: Fast and accurate defocus estimation from electron micrographs. *J. Struct. Biol.* **192**, 216–221 (2015).
10. T. Wagner, *et al.*, Two particle-picking procedures for filamentous proteins: SPHIRE-crYOLO filament mode and SPHIRE-STRIPER. *Acta Crystallogr. Sect. Struct. Biol.* **76**, 613–620 (2020).
11. J. Schindelin, *et al.*, Fiji: an open-source platform for biological-image analysis. *Nat. Methods* **9**, 676–682 (2012)
12. D. Emin, *et al.*, Small soluble α-synuclein aggregates are the toxic species in Parkinson’s disease. *Nat. Commun.* **13**, 5512 (2022).
13. C. Galvagnion, et al., Lipid vesicles trigger α-synuclein aggregation by stimulating primary nucleation. Nat. Chem. Biol. 11, 229–234 (2015).
14. N. N. Ninkina, *et al.*, Organization, expression and polymorphism of the human Persyn gene. *Hum. Mol. Genet.* **7**, 1417–1424 (1998).
15. T. Lee, L. Luo, Mosaic analysis with a repressible cell marker for studies of gene function in neuronal morphogenesis. *Neuron* **22**, 451–461 (1999).
16. J. W. Gargano, I. Martin, P. Bhandari, M. S. Grotewiel, Rapid iterative negative geotaxis (RING): a new method for assessing age-related locomotor decline in Drosophila. *Exp. Gerontol.* **40**, 386–395 (2005).
17. N. Ninkina, et al., Neurons Expressing the Highest Levels of γ-Synuclein Are Unaffected by Targeted Inactivation of the Gene. Mol. Cell. Biol. 23, 8233–8245 (2003).

18. G. Fusco, *et al.*, Structural basis of membrane disruption and cellular toxicity by alpha-synuclein oligomers. *Science* **358**, 1440-+ (2017).

19. P. Alam, L. Bousset, R. Melki, D. E. Otzen, α-synuclein oligomers and fibrils: a spectrum of species, a spectrum of toxicities. *J. Neurochem.* **150**, 522–534 (2019).

20. S. T. Kumar, S. Donzelli, A. Chiki, M. M. K. Syed, H. A. Lashuel, A simple, versatile and robust centrifugation-based filtration protocol for the isolation and quantification of α-synuclein monomers, oligomers and fibrils: Towards improving experimental reproducibility in α-synuclein research. *J. Neurochem.* **153**, 103–119 (2020).

21. K. Fujiwara, H. Toda, M. Ikeguchi, Dependence of α-helical and β-sheet amino acid propensities on the overall protein fold type. *BMC Struct. Biol.* **12**, 18 (2012).

22. A. C. Tsolis, N. C. Papandreou, V. A. Iconomidou, S. J. Hamodrakas, A consensus method for the prediction of 'aggregation-prone' peptides in globular proteins. *PLOS ONE* **8**, e54175 (2013).

23. V. Muñoz, L. Serrano, Elucidating the folding problem of helical peptides using empirical parameters. *Nat. Struct. Biol.* **1**, 399–409 (1994).
